# LINC00659 cooperated with ALKBH5 to accelerate gastric cancer progression by stabilising JAK1 mRNA in an m^6^A‐YTHDF2‐dependent manner

**DOI:** 10.1002/ctm2.1205

**Published:** 2023-03-02

**Authors:** Yuan Fang, Xi Wu, Yunru Gu, Run Shi, Tao Yu, Yutian Pan, Jingxin Zhang, Xinming Jing, Pei Ma, Yongqian Shu

**Affiliations:** ^1^ Department of Oncology The First Affiliated Hospital of Nanjing Medical University Nanjing People's Republic of China; ^2^ Department of General Surgery Affiliated People's Hospital of Jiangsu University Zhenjiang Clinic School of Nanjing Medical University Zhenjiang People's Republic of China; ^3^ Jiangsu Key Laboratory of Cancer Biomarkers Prevention and Treatment Collaborative Innovation Center for Cancer Personalized Medicine Nanjing Medical University Nanjing People's Republic of China

## Abstract

**Background:**

N6‐methyladenosine (m^6^A) RNA modification is known as a common epigenetic regulation form in eukaryotic cells. Emerging studies show that m^6^A in noncoding RNAs makes a difference, and the aberrant expression of m^6^A‐associated enzymes may cause diseases. The demethylase alkB homologue 5 (ALKBH5) plays diverse roles in different cancers, but its role during gastric cancer (GC) progression is not well known.

**Methods:**

The quantitative real‐time polymerase chain reaction, immunohistochemistry staining and western blotting assays were used to detect ALKBH5 expression in GC tissues and human GC cell lines. The function assays in vitro and xenograft mouse model in vivo were used to investigate the effects of ALKBH5 during GC progression. RNA sequencing, MeRIP sequencing, RNA stability and luciferase reporter assays were performed to explore the potential molecular mechanisms involved in the function of ALKBH5. RNA binding protein immunoprecipitation sequencing (RIP‐seq), RIP and RNA pull‐down assays were performed to examine the influence of LINC00659 on the ALKBH5–JAK1 interaction.

**Results:**

ALKBH5 was highly expressed in GC samples and associated with aggressive clinical features and poor prognosis. ALKBH5 promoted the abilities of GC cell proliferation and metastasis in vitro and in vivo. The m^6^A modification on JAK1 mRNA was removed by ALKBH5, which resulted in the upregulated expression of JAK1. LINC00659 facilitated ALKBH5 binding to and upregulated JAK1 mRNA depending on an m^6^A‐YTHDF2 manner. Silencing of ALKBH5 or LINC00659 disrupted GC tumourigenesis via the JAK1 axis. JAK1 upregulation activated the JAK1/STAT3 pathway in GC.

**Conclusion:**

ALKBH5 promoted GC development via upregulated JAK1 mRNA expression mediated by LINC00659 in an m^6^A‐YTHDF2‐dependent manner, and targeting ALKBH5 may be a promising therapeutic method for GC patients.

## Graphical abstract

- LINC00659 recruits ALKBH5 to form a complex that binds to JAK1 and decreases the m^6^A levels of JAK1 mRNA.
- The removal of JAK1 m^6^A modifications promotes the RNA stability of JAK1 that upregulates expression of JAK1.
- Upregulated JAK1‐activated STAT3 and JAK1/STAT3 pathway promote GC progression.

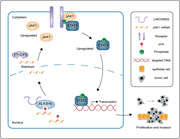

## 1 INTRODUCTION

Gastric cancer (GC) is one of the most frequent cancers worldwide and is easy to diagnose due to its great features.^1^, ^2^ Unfortunately, most patients are diagnosed in an advanced stage with multiple metastases when opportunities for radical surgery are lost. Because the 5‐year survival rate for GC is less than 10%,^3^, ^4^, ^5^ it is necessary to further elucidate the mechanisms of GC progression to develop novel therapeutic targets.

N6‐methyladenosine (m^6^A) refers to the methylation of adenosine at position 6, and it is one of the most common and best‐characterised modifications.^6^, ^7^ Abnormal m^6^A modification may influence noncoding RNA expression, which affects the progression of cancer.^8^ As we known, m^6^A modification can be deposited by the m^6^A methyltransferase METTL3–METTL14–WTAP complex^9^, ^10^, ^11^ and reversibly removed by two demethylases, fat mass and obesity‐associated protein (FTO) and alkB homologue 5 (ALKBH5).^12^, ^13^ It is also recognised by special proteins named ‘readers’, including the YT521‐B homology (YTH) domain‐containing family (YTHDFs) and the insulin‐like growth factor 2 mRNA‐binding protein (IGF2BPs) family.^14^ Emerging evidence has revealed that the aberrant expression of m^6^A‐associated enzymes, such as ALKBH5, may lead to tumourigenesis, including lung cancer, pancreatic cancer and hepatocellular carcinoma, and these enzymes are promising therapeutic targets.^15^, ^16^, ^17^, ^18^, ^19^ However, further research is required to explain how ALKBH5 mediates GC progression.

The role of ALKBH5 in GC is controversial. A previous study indicated that ALKBH5 was a cancer‐promoting gene in GC.^20^ However, Hu et al.^21^ showed that ALKBH5 suppressed the invasion of GC via the ALKBH5–PKMYT1–IGF2BP3 axis. Therefore, the role of this enzyme remains controversial, possibly due to the different cancer models, and the mechanism of action of ALKBH5 in GC needs further investigation.

The Janus kinase (JAK)–STAT pathway plays an essential role in cancer development. JAK1 is a member of the JAK family that activates the free STAT molecule distributed in the cytoplasm.^22^ A number of seminal papers showed that the JAK/STAT pathway promoted the survival and proliferation of tumour cells and a variety of other cancer‐related hallmarks over the last decade.^23^ JAK1/STAT3 pathway activation significantly promotes GC cell proliferation and invasion.^24^

The m^6^A modification regulates the formation and function of noncoding RNAs. Noncoding RNAs also have regulatory effects on m^6^A modifications.^25^ Zhang et al.^15^ found that the long noncoding RNA a long noncoding RNA antisense to FOXM1 (FOXM1‐AS) promoted the interaction between ALKBH5 and FOXM1, which contributed to the subsequent demethylation of FOXM1.

The present study found an oncogenic role of ALKBH5 in GC proliferation and metastasis. We also found that ALKBH5 caused m^6^A removal in JAK1 mRNA with the help of LINC00659, which enhanced JAK1 mRNA stability and contributed to the upregulation of JAK1 in GC. In summary, our work demonstrated that LINC00659 functioned in the modification of ALKBH5‐mediated JAK1 mRNA, which was involved in GC progression, and these results provide a new treatment target for GC.

## 2 MATERIALS AND METHODS

### 2.1 Data acquisition, preprocessing and bioinformatic analysis

To evaluate ALKBH5 mRNA expression in GC, transcripts per million values of ALKBH5 were obtained from 414 GC samples and 36 adjacent normal tissues uploaded to The Cancer Genome Atlas (TCGA) portal (https://portal.gdc.cancer.gov/).

We acquired the transcriptome profiling data and corresponding survival outcomes of 300 GC samples from the Asian Cancer Research Group (ACRG) study for further analysis (downloaded from Gene Expression Omnibus [GSE62254]), and patients lost to follow‐up were excluded during survival analysis. We used the survminer R package to determine the optimal cutoff value. The probe IDs were mapped to gene symbols based on the GPL570 platform annotation file (Affymetrix Human Genome U133 Plus 2.0 Array), the maximal probe measurement was used to determine the final gene expression of ALKBH5 (probe ID: 234302_s_at). The RNA sequencing (RNA‐seq) and microarray data used in this study were normalised and log2‐transformed prior to use.

In order to explore the potential roles of ALKBH5 in clinical GC samples, we extracted the expression matrix of tumour samples with the lowest and highest ALKBH5 expression from ACRG using the decile method and identified 541 significantly upregulated genes using the Limma R package (false discovery rate [FDR] <0.05). Next, these genes were used to perform Gene Ontology (GO) enrichment analysis, and cancer hallmark‐related pathways are displayed in a Circos plot.

### 2.2 Specimens and cell culture

We collected the GC tissues and adjacent normal gastric tissues from patients with GC. All the patients with GC needed surgical treatment and were hospitalised in the Affiliated People's Hospital of Jiangsu University. The study received ethical approval from Nanjing Medical University (2018‐SRFA‐074) and Jiangsu University Affiliated People's Hospital (K20180016Y), which was implemented in accordance with the Helsinki Declaration of Principles. All gastric tissues and paired adjacent tissues were surgically removed and used for RNA and protein extraction and immunohistochemistry (IHC) analysis. Details of the antibodies used for IHC are shown in Table S2. To calculate the total score for ALKBH5 or JAK1 immunostaining, we first assessed the proportion of positively stained tumour cells (PP, 0–4) and the staining intensity (SI, 0–3) according to following criteria. We scored the PP in four categories: 0 (0%), 1 (5%–25%), 2 (25%–50%) and 3 (>51%). SI was scored on a scale of 0–3 (0, negative; 1, weak; 2, moderate; 3, strong). We multiplied the SI and PP scores to obtain a staining score, ranging from 0 to 9. There were two groups of positive levels of IHC staining: the low ALKBH5/JAK1 (0–3) group and high ALKBH5/JAK1 group (4–9). The human GC cell lines AGS, BGC‐823, MGC‐803, SGC‐7901 and normal gastric epithelial cell line GES‐1 were procured from Shanghai Cell Bank Library (Shanghai, China) and cultured in RPMI 1640 (BI, Israel) supplemented with 10% foetal bovine serum (BI) at 37°C under 5% CO_2_ conditions.

### 2.3 Cell proliferation and colony formation assay

Processes of the colony formation assay and cell counting kit‐8 (CCK8) assays are described in a previously published article.^26^

### 2.4 Transwell assay

Transwell assays were performed in accordance with a previously published article.^26^

### 2.5 RNA pull‐down assay

The RNAmax‐T7 Kit (RiboBio) and RNeasy Mini Kit (QIAGEN) were used to obtain and purify biotinylated RNAs. An RNA protein pull‐down kit from Pierce (Thermo, USA) was used for pull‐down assays. Biotinylated RNAs were rotated with streptavidin agarose beads for 2 h. BGC‐823 cells were lysed using IP Lysis Buffer (Pierce). After several washes, the mixture of the lysate, protease/phosphatase inhibitor cocktail, RNase inhibitor and streptavidin agarose beads was rotated at 4°C for 1 h. After washing, protein was obtained from beads after 30 min of rotation in elution buffer at 37°C.

### 2.6 RNA binding protein immunoprecipitation assay

Antibodies against ALKBH5 (Abcam, #ab195377) and YTHDF2 (Abcam, #ab220163) were used in RNA binding protein immunoprecipitation (RIP) assays. The detailed method of RIP assay is described in a previously published paper.^27^

### 2.7 RNA stability

We treated different groups of GC cells with actinomycin D (2 µg/mL) when cells reached 40%–50% confluence and harvested for RNA extraction after 0, 6 and 12 h of treatment.

### 2.8 Animal studies

Nanjing Medical University's Committee on Ethics for Animal Experiments approved the experimental procedures (IACUC‐1706007). All mice used were on the BALB/c nude background. The Nanjing Medical University Animal Center provided 6–8‐week‐old female BALB/c nude mice for our research. BGC‐823 (3 × 10^6^) cells that stably expressed or silenced ALKBH5 and LINC00659 and their paired control cells were injected into the left side of each mouse. Every 5 days, the tumour volume was measured, mice were euthanised 20 days after injection and the weights were measured. Tumours were used for RNA extraction, western blotting (WB) and IHC assays.

For cell metastasis experiments in vivo, BGC‐823 (3 × 10^6^) cells that stably overexpressed or silenced ALKBH5 and LINC00659 and their paired control cells were injected through tail vein. All mice were euthanised, and metastatic organs were removed after 56 days of injection. Their lungs and livers were collected for further analysis (such as WB, haematoxylin and eosin [H&E] staining and IHC staining).

More details of the remaining materials and methods are available in Supporting Information.

## 3 RESULTS

### 3.1 ALKBH5 is overexpressed in GC tissues and associated with poor survival

**FIGURE 1:**
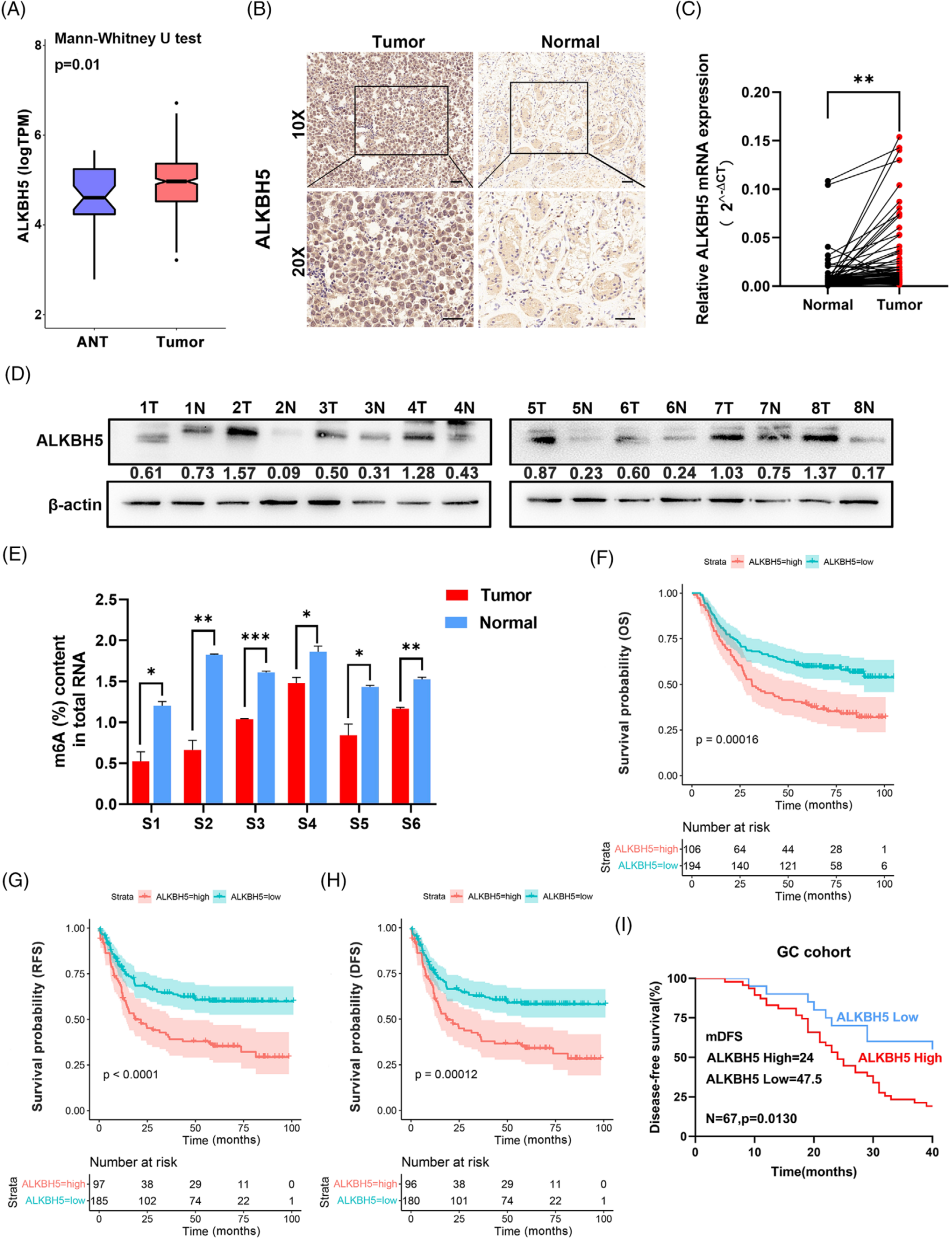
AlkB homologue 5 (ALKBH5) is highly expressed in gastric cancer (GC) tissues and is associated with poor prognosis. (A) Whole TCGA (The Cancer Genome Atlas) GC database demonstrated that ALKBH5 was upregulated in 414 GC tumour samples compared with 37 normal samples. (B) Representative immunohistochemistry (IHC) staining showed ALKBH5‐positive stained cells in GC tissues and matched adjacent gastric tissues (scale bars = 50 µm). (C) The levels of ALKBH5 expression in GC and matched adjacent gastric tissues were detected by quantitative real‐time polymerase chain reaction (qRT‐PCR) (*n* = 67). The PCR results were normalised to the expression of β‐actin. (D) ALKBH5 protein levels were detected in GC tissues (T) and matched adjacent gastric tissues (N) by western blotting (*n* = 8). Relative protein levels were normalised to β‐actin. (E) The N6‐methyladenosine (m^6^A) RNA levels in six GC tissues and matched adjacent gastric tissues were checked by colorimetric ELISA assay using the m^6^A RNA methylation quantification kit. (F–G) Data from the Asian Cancer Research Group (ACRG) study showed that OS, RFS and DFS of patients with ALKBH5 high expression were shorter. (I) Kaplan–Meier analysis revealed DFS in GC patients based on the relative ALKBH5 expression (ALKBH5‐High, *n* = 48; ALKBH5‐Low, *n* = 19). This analysis was based on our IHC cohort. *p < .05; ^**^ *p* < .01; ^***^ *p* < .001.

To examine whether ALKBH5 was dysregulated in GC tissues, we analysed the entire TCGA GC database, and the results demonstrated that ALKBH5 was highly expressed in 414 GC tumour samples compared to 37 normal gastric mucosa samples (Figure 1A). We performed quantitative real‐time polymerase chain reaction (qRT‐PCR) and IHC staining in 67 pairs of GC tissues and normal mucosa, and we found that ALKBH5 was present in GC tissues in much greater amounts than in normal tissues (Figure 1B,C). WB assays also showed high expression levels of ALKBH5 in GC tissues (seven of eight) (Figure 1D).

According to the level of ALKBH5 in 67 pairs of GC tissues, we divided the samples into ALKBH5‐High (*n* = 48) and ALKBH5‐Low (*n* = 19) groups. Subsequently, we surprisingly observed that ALKBH5 expression level positively correlated with more aggressive clinicopathological characteristics (such as histological differentiation, invasion depth, Tumor, Node, Metastasis (TNM) stage and lymphatic metastasis) (Table 1). The negative correlation between ALKBH5 expression and global m^6^A levels was confirmed in six fresh human GC tissues (Figure 1E). Transcriptome profiling data of 300 GC samples and corresponding survival outcomes obtained from the ACRG study showed that the Overall survival (OS), Recurrence‐Free‐Survival (RFS) and Disease‐Free‐Survival (DFS) of ALKBH5 high group were shorter (Figure 1F–H). Similarly, our GC cohort data also indicated that patients in the ALKBH5‐High group had shorter DFS, as shown by Kaplan–Meier survival curves (Figure 1I). Collectively, our findings revealed that ALKBH5 was highly present in GC and could serve as a prognostic biomarker for GC patients.

**TABLE 1:** Correlation between alkB homologue 5 (ALKBH5) expression and clinicopathological characteristics of gastric cancer (GC).

	ALKBH5 expression		
Clinical parameter	High (*n* = 48)	Low (*n* = 19)	*χ* ^2^ test (*p*‐value)
Age (years)			.332387
<60	12	7	
>60	36	12	
Gender			.403892
Male	33	15	
Female	15	4	
Size			.365532
<5 cm	31	10	
>5 cm	17	9	
Histologic differentiation			.017747*
Moderate	13	11	
Poor	35	8	
Invasion depth			.025682*
T1/T2	16	12	
T3/T4	32	7	
TNM stages			.029856*
I/II	10	9	
III/IV	38	10	
Lymphatic metastasis			.029945*
Yes	36	9	
No	12	10	

* *p* < .05.

### 3.2 ALKBH5 promotes GC cell proliferation and metastasis in vitro and in vivo

Firstly, we evaluated the expression of ALKBH5 in GC cell lines and GES1 normal gastric epithelial cell lines (Figure S1a,b). Because ALKBH5 was highly overexpressed in BGC‐823 and MGC‐803 cells, we chose these cells for further study. We stably silenced ALKBH5 (sh‐NC group and shALKBH5 group) and overexpressed ALKBH5 (Negative control (NC) group and ALKBH5 group) in BGC‐823 and MGC‐803 cells, which were confirmed using qPCR and WB assays (Figure S1c–f). The abundance of m^6^A was colorimetrically measured in GC cells using ELISA. We found that ALKBH5 significantly decreased the m^6^A level in BGC‐823 and MGC‐803 cells (Figure S1g,h).

**FIGURE 2:**
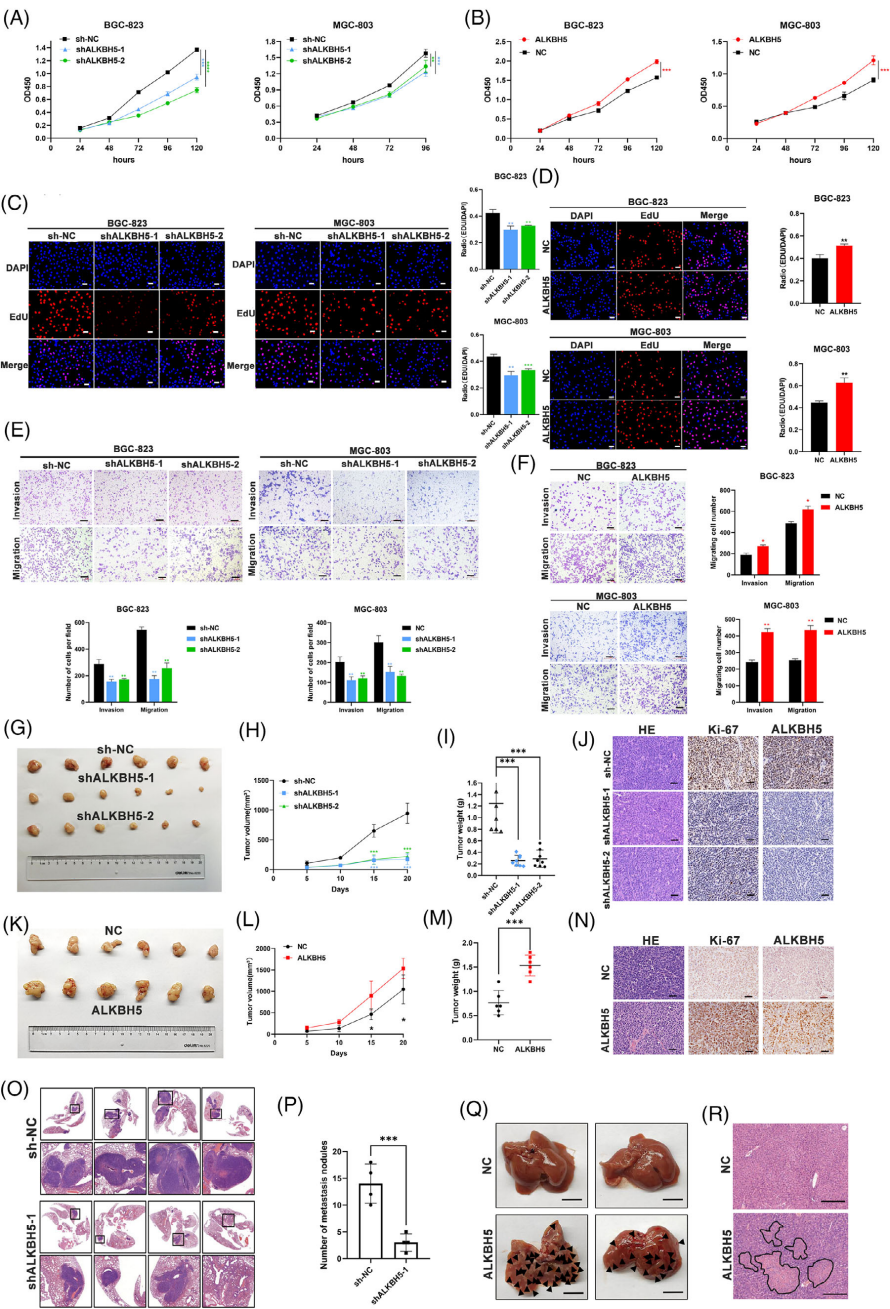
AlkB homologue 5 (ALKBH5) promotes gastric cancer (GC) proliferation and metastasis in vivo and in vitro. (A) Cell counting kit‐8 (CCK8) assays were conducted to determine cell proliferation of BGC‐823 and MGC‐803 cells with stable silenced ALKBH5. (B) CCK8 assays were performed to determine cell proliferation of BGC‐823 and MGC‐803 cells with stable ALKBH5 overexpression. (C and D) EdU assays were conducted to determine cell proliferation of BGC‐823 and MGC‐803 cells with stably silenced ALKBH5 (C) and overexpressed ALKBH5 (D) (scale bars = 100 mm). (E and F) Cell migration and invasion assays of BGC823 cells and MGC‐803 cells were performed by Transwell assays after knockdown (E) or overexpression (F) of ALKBH5 (scale bars = 100 mm). (G) The images of dissected tumours from BGC‐823 cells stably transfected with sh‐NC and sh‐ALKBH5. (H and I) Tumour weights and sizes are represented as means of tumour weights (I)/sizes (H) ± standard deviation (SD). (J) Tumour tissue samples were immunostained for haematoxylin and eosin (H&E), Ki‐67 and ALKBH5 (magnification, ×400, scale bars = 10 µm). (K–M) Overexpression of ALKBH5 promoted the growth of xenografted tumours. BGC‐823 cells with NC or ALKBH5 overexpression were subcutaneously injected into nude mice. (N) Representative immunohistochemistry results and quantification of Ki‐67 and ALKBH5‐positive staining in tumours (magnification, ×400, scale bars = 10 µm). (O and P) Visualisation of the entire lung, and H&E‐stained lung sections. Knockdown of ALKBH5 in BGC‐823 cells markedly suppressed GC lung metastasis in nude mice (*n* = 4) (scale bars = 200 mm). (Q) Representative images of the metastatic nodes in the livers (scale bars = 200 mm). (R) H&E‐stained liver sections (scale bars = 200 µm). ^*^ *p* < .05; ^**^ *p* < .01; ^***^ *p* < .001.

GO analysis was performed using 541 significantly upregulated genes of tumour samples with the lowest and highest ALKBH5 expression from ACRG. Cancer hallmark‐related pathways, including cancer proliferation and metastasis, are shown in the Circos plot, which indicated a close association of ALKBH5 and cancer proliferation and metastasis (Figure S1i). According to the results of CCK8, EdU and colony formation assays, the ability of GC cell proliferation was inhibited by ALKBH5 knockdown in vitro (Figures 2A,C and S1j). We observed that knockdown of ALKBH5 inhibited the invasion and migration of GC cells by performing Transwell assays (Figure 2E). In contrast, overexpressing ALKBH5 benefited GC cell growth in vitro (Figures 2B,D and S1k). Transwell assays showed that overexpressing ALKBH5 enhanced the abilities of GC cells to migrate and invade in vitro compared to the NC groups (Figure 2F). Based on Hu et al.’s study,^21^ AGS and NCI‐N87 cells were also tested for colony formation, CCK8, and Transwell migration after overexpressing or knocking down ALKBH5. The expression of ALKBH5 in each group of AGS and NCI‐N87 cells was detected using WB assays (Figure S2a,b). The same results of functional biology in AGS and NCI‐N87 cells were obtained as in MGC‐803 and BGC‐823 cells (Figure S2c–j).

To investigate the function of ALKBH5 in vivo, we established a mouse xenograft model by subcutaneously injecting nude mice with BGC‐823–shALKBH5, BGC‐823–ALKBH5 and the corresponding control cells. Consistent with the in vitro results, shALKBH5‐1 and shALKBH5‐2 tumours were significantly smaller and lighter than control tumours (Figure 2G‐I). IHC assays showed decreased expression of Ki‐67 (a key biomarker in tumour growth) and suppressed expression of ALKBH5 in the shALKBH5‐1 and shALKBH5‐2 groups (Figure 2J), which were consistent with the WB results (Figure S1l). Tumours in the BGC‐823–ALKBH5 group had a larger volume heavier weight (Figure 2K‐M), and higher expression of Ki‐67 and ALKBH5 (Figures 2N and S1m).

We explored the metastasis potential of ALKBH5 in vivo by injecting BGC‐823 cells with stable knockdown or overexpression of ALKBH5 and control cells into nude mice via the tail vain. H&E staining assays showed that the number of metastatic nodes in lung or liver was much lower in the ALKBH5‐downregulated group (Figure 2O,P). However, ALKBH5 overexpression resulted in more lung and liver metastasis (Figures 2Q,R and S1n).

Collectively, all the evidence suggested that ALKBH5 acted as a cancer‐promoting regulator in GC in vitro and in vivo.

### 3.3 JAK1 was the downstream target of ALKBH5

**FIGURE 3:**
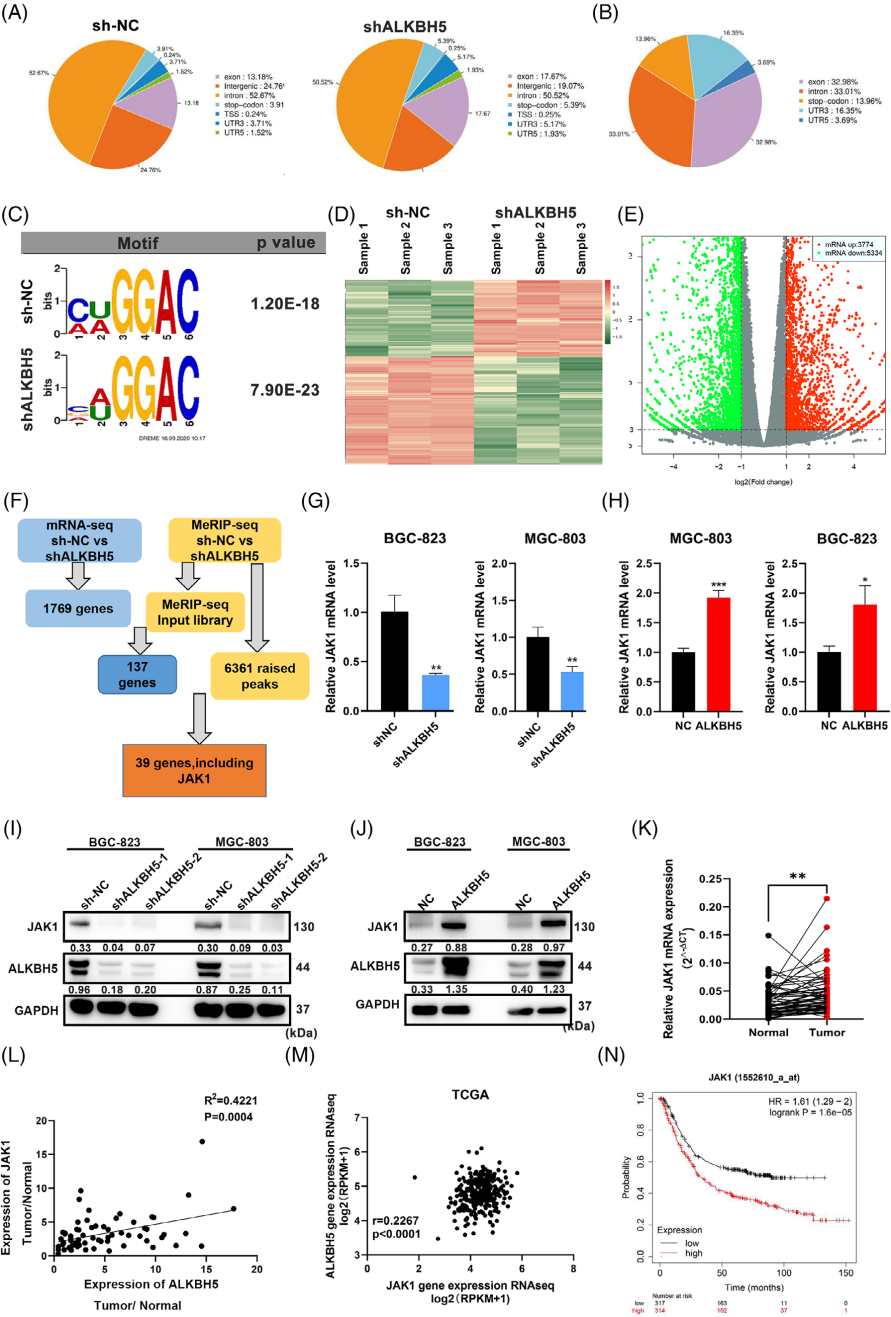
JAK1 was the downstream target of alkB homologue 5 (ALKBH5). (A) The pattern of N6‐methyladenosine (m^6^A) peak distribution was slightly changed after ALKBH5 knockdown according to MeRIP‐seq data. (B) The locations of m^6^A peaks from 6598 transcripts. (C) The m^6^A motif detected by the MEME motif analysis with MeRIP‐seq data. (D) Heatmap of differentially expressed mRNA identified by RNA‐seq after ALKBH5 knockdown. (E) RNA‐seq results identified 3774 upregulated mRNAs and 5334 downregulated mRNAs in ALKBH5‐knockdown BGC‐823 cells. (F) Flowchart demonstrates the selection process of the downstream target of ALKBH5. (G) The RNA expression levels of JAK1 in ALKBH5‐deficient BGC‐823 cells and MGC‐803 cells were examined by quantitative real‐time polymerase chain reaction (qRT‐PCR). (H) The RNA expression levels of JAK1 in ALKBH5‐overexpressing BGC‐823 cells and MGC‐803 cells were examined by qRT‐PCR. (I) The protein expression levels of JAK1 in ALKBH5‐deficient BGC‐823 cells and MGC‐803 cells were examined by western blotting. Relative protein levels were normalised to GAPDH. (J) The protein expression levels of JAK1 in ALKBH5‐overexpressing BGC‐823 cells and MGC‐803 cells were examined by western blotting. Relative protein levels were normalised to GAPDH. (K) The levels of JAK1 expression in gastric cancer (GC) and matched adjacent gastric tissues were detected by qRT‐PCR (*n* = 67). (L) ALKBH5 expression was positively correlated with JAK1 expression in GC cohort (linear regression). (M) ALKBH5 expression was positively correlated with JAK1 expression in TCGA database. (N) Online bioinformatics tool Kaplan–Meier plotter found that GC patients with increased expression of JAK1 had significantly reduced OS. ^*^ *p* < .05; ^**^ *p* < .01; ^***^ *p* < .001.

To examine the specific mechanism of ALKBH5 in GC tumourigenesis, we performed MeRIP sequencing (MeRIP‐seq) and RNA‐seq in ALKBH5‐knockdown and control BGC‐823 cells. MeRIP‐seq revealed that the m^6^A peaks of 6361 transcripts were increased after ALKBH5 knockdown (fold change >2; FDR <0.05), which indicated a global gain of m^6^A methylation in mRNA transcripts with ALKBH5 knockdown. A minor change in the pattern of m^6^A peak distribution was detected. Most m^6^A peaks were located in the exon (32.98%) and intron (33.01%) areas (Figure 3A). These differential m^6^A peaks of transcripts in the two groups are shown in Figure 3B. The m^6^A consensus motif GGAC was highly enriched in ALKBH5‐knockdown and control BGC‐823 cells (Figure 3C). RNA‐seq showed that 9108 transcripts were changed in ALKBH5‐knockdown BGC‐823 cells, among which 3774 transcripts were increased (fold change >2), and 5334 transcripts were reduced compared to the control (fold change <0.5; FDR <0.05) (Figure 3D,E). These transcripts were from 1769 genes.

A total of 137 genes were shared between the RNA‐seq data and MeRIP‐seq input library data. We overlapped 137 genes with 6361 transcripts, m^6^A peaks were increased after ALKBH5 knockdown, and there were 39 mutual genes. These genes were enriched in different signalling pathways, such as transforming growth factor‐beta receptor signalling and interleukin (IL)‐4 and IL‐13 signalling. JAK1 and FOS were found in all signalling pathways (Table S3). We further examined the expression of JAK1 and FOS in ALKBH5‐knockdown GC cells and found that JAK1 was decreased in BGC‐823 and MGC‐803 cells after ALKBH5 knockdown (Figure S3a,b). Therefore, we selected JAK1 for further study (Figure 3F). We examined the expression of JAK1 in ALKBH5‐knockdown and ALKBH5‐overexpressing cells using qRT‐PCR. We observed significant downregulation of JAK1 transcripts in ALKBH5‐knockdown cells, and ALKBH5 overexpression increased JAK1 mRNA in BGC‐823 and MGC‐803 cells (Figure 3G,H). These results were further confirmed using WB (Figure 3I,J). Therefore, ALKBH5 regulated the expression of JAK1. We examined the expression of other members of the JAK family, such as JAK2 and JAK3, using qRT‐PCR and found no difference after ALKBH5 knockdown in GC cells (Figure S3c,d). We also performed MeRIP‐qPCR. After silencing ALKBH5, JAK2/3 mRNA was enriched with an anti‐m^6^A antibody but was not different from the control group (Figure S3e,f). Therefore, we assumed that JAK2 and JAK3 mRNAs are not targets of ALKBH5.

We examined the relationship between JAK1 and GC by comparing the expression of JAK1 in 67 pairs of normal gastric mucosa and GC tissues (Figure 3K). An increase in JAK1 was observed in GC tissues, and its expression positively correlated with ALKBH5 (*R* ^2^ = 0.4221, *p* = .0004) (Figure 3L). The online TCGA database verified these results (Figure 3M). Figures 3N and S3g show that GC patients with increased JAK1 expression had significantly shorter OS and Progression‐Free‐Survival (PFS) than those with low JAK1 expression group using the online bioinformatics website Kaplan–Meier plotter.

### 3.4 ALKBH5 removes m^6^A modifications from JAK1 mRNA and maintains JAK1 mRNA stability in a YTHDF2‐dependent manner

**FIGURE 4:**
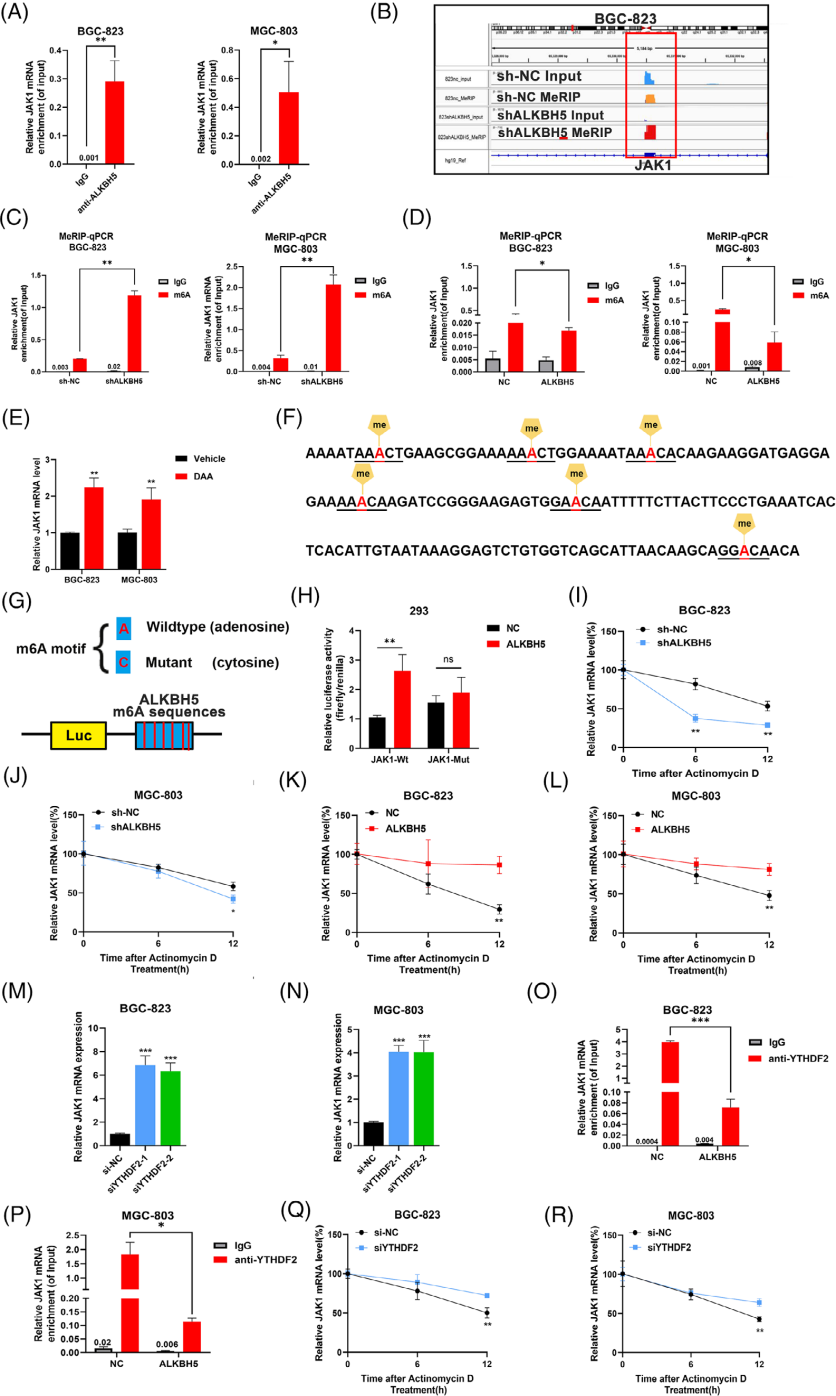
AlkB homologue 5 (ALKBH5) removes N6‐methyladenosine (m^6^A) modifications of JAK1 mRNA and maintains JAK1 mRNA stability in an YTHDF2‐dependent manner. (A) RNA binding protein immunoprecipitation (RIP)‐qPCR assays confirmed that JAK1 mRNA binding to ALKBH5. (B) m^6^A modification of JAK1 mRNA was visualised by Integrative Genomics Viewer (IGV) software after ALKBH5 knockdown. The significantly increased m^6^A peak is indicated by red rectangles. (C) The m^6^A abundances on JAK1 mRNA after ALKBH5 knockdown were checked by MeRIP‐qPCR. m^6^A on JAK1 mRNA was increased after ALKBH5 knockdown. (D) The m^6^A abundances on JAK1 mRNA after ALKBH5 overexpression were checked by MeRIP‐qPCR. m^6^A on JAK1 mRNA was decreased after ALKBH5 knockdown. (E) BGC‐823 and MGC‐803 cells were treated with a global methylation inhibitor (DAA), leading to the upregulation of JAK1 mRNA levels. (F) Six potential m^6^A sites were found on the sequences of raised m^6^A peaks of JAK1 transcripts after ALKBH5 knockdown according to MeRIP‐seq data. (G) All adenosines (A) were replaced with cytosines (C) in RRACH motif. These partial sequences of JAK1 mRNA were inserted with wild‐type or mutated m^6^A sites into luciferase reporter plasmids. (H) Relative luciferase activity (firefly/Renilla activity) of the wild‐type or mutant JAK1 luciferase reporter in 293 cells with ALKBH5 overexpression and the negative control. The results were normalised to the wild‐type with negative control group. (I‐L) ALKBH5 on mRNA stability of JAK1 was detected by use of actinomycin D (2 µg/mL) on BGC‐823 and MGC‐803 cells with stable ALKBH5 knockdown or overexpression. The results were normalised to the data of 0 h. (M and N) Knocking down YTHDF2 significantly increased the mRNA expression of JAK1 detected by quantitative real‐time polymerase chain reaction (qRT‐PCR). (O and P) RIP‐qPCR assays detected the change of binding capacity between JAK1 and YTHDF2 after ALKBH5 overexpression. (Q and R) YTHDF2 on mRNA stability of JAK1 was detected by use of actinomycin D (2 µg/mL) on GC cells with YTHDF2 knockdown. ^*^ *p* < .05; ^**^ *p* < .01; ^***^ *p* < .001.

Because ALKBH5 influenced JAK1 mRNA levels, RIP assays in GC cells were performed, and the results showed that more JAK1 mRNA was enriched with anti‐ALKBH5 antibodies than immunoglobulin (IgG), which indicated direct binding between ALKBH5 and JAK1 (Figure 4A). RNA–protein colocalisation of JAK1 mRNA with ALKBH5 was performed, which indicated that ALKBH5 binds to JAK1 mRNA in the nucleus (Figure S4a). Because ALKBH5 is an m^6^A demethylase, we presumed that ALKBH5 regulated the expression of JAK1 by erasing m^6^A modifications. As shown in the MeRIP‐seq data, the m^6^A abundance of JAK1 mRNA was notably increased after ALKBH5 knockdown, and this peak was located in chr1, 65330528–65330698, which is the coding sequence of JAK1 (Figure 4B). The RRACH (R=G or A, H=A, C or U) sequences are known as the m^6^A binding sites in previous studies,^28^ and the GGAC motif was found in JAK1 mRNA using MeRIP‐seq analysis. We performed MeRIP‐qPCR in BGC‐823 and MGC‐803 cells. After silencing ALKBH5, more JAK1 mRNA was enriched with the m^6^A antibody, which suggested that the m^6^A abundance of JAK1 mRNA was increased (Figure 4C). Consistently, overexpressing ALKBH5 caused the removal of m^6^A in JAK1 mRNA (Figure 4D). We treated GC cells with the nonspecific methylation inhibitor 3‐deazaadenosine (DAA) and found that JAK1 mRNA expression increased significantly after administration of DAA (Figure 4E).

To verify the binding site between ALKBH5 and JAK1 mRNA, we found six potential m^6^A sites on the sequence (chr1, 65330528–65330698) of increased m^6^A peaks of JAK1 transcripts after ALKBH5 knockdown according to MeRIP‐seq data. This sequence was located in the exon of JAK1 (Figure 4F). We replaced all adenosines (A) with cytosines (C) in the RRACH motif and inserted a partial sequence of JAK1 mRNA with wild‐type (Wt) or mutant m^6^A sites into luciferase reporter plasmids (Figure 4G). ALKBH5 increased relative luciferase activity in the JAK1‐Wt group, but the JAK1‐Mut group had no effect on luciferase activity (Figure 4H).

In summary, ALKBH5 regulated the JAK1 expression level in an m^6^A‐dependent manner.

To further investigate the effect of m^6^A modification on JAK1 mRNA, we treated GC cells with actinomycin D, which restrained the transcription of RNA. JAK1 mRNA had a faster decay rate when ALKBH5 was silenced, and the mRNA was more stable with ALKBH5 overexpression, which indicated that m^6^A modification affected the stability of JAK1 mRNA (Figure 4I‐L).

YTHDF2 binds to the attenuation sites of mRNAs to cause the decay of certain mRNAs.^29^, ^30^ A recent study reported that YTHDF2 recognised m^6^A modification and targeted thousands of transcripts, including JAK1.^30^ To evaluate the effect of YTHDF2 on JAK1 mRNA, we transferred two small interfering RNAs (siRNAs) to silence the expression of YTHDF2 in GC cells. YTHDF2 knockdown significantly increased the mRNA level of JAK1 in GC cell lines (Figure 4M,N). We performed RIP‐qPCR assays in ALKBH5‐overexpressing GC cells. Overexpressing ALKBH5 decreased the enrichment between YTHDF2 and JAK1 mRNA, which indicated that YTHDF2 recognised the m^6^A modification of JAK1 mRNA and influenced its expression (Figure 4O,P). Knockdown of YTHDF2 stabilised JAK1 mRNA (Figure 4Q,R). Furthermore, we found that YTHDF2 deletion rescued the decrease in JAK1 levels caused by ALKBH5 knockdown in GC cells (Figure S4b).We have blocked YTHDF2 in the proliferation, invasion and migration of ALKBH5 knockdown cells in vitro and found that YTHDF2 deletion rescued the decrease in ALKBH5 knockdown‐mediated function in GC (Figure S4c–e).Taken together, our findings indicated that ALKBH5‐mediated m^6^A modification influenced JAK1 mRNA stability in a YTHDF2‐dependent manner.

### 3.5 LINC00659 facilitates ALKBH5 binding to JAK1 mRNA

**FIGURE 5:**
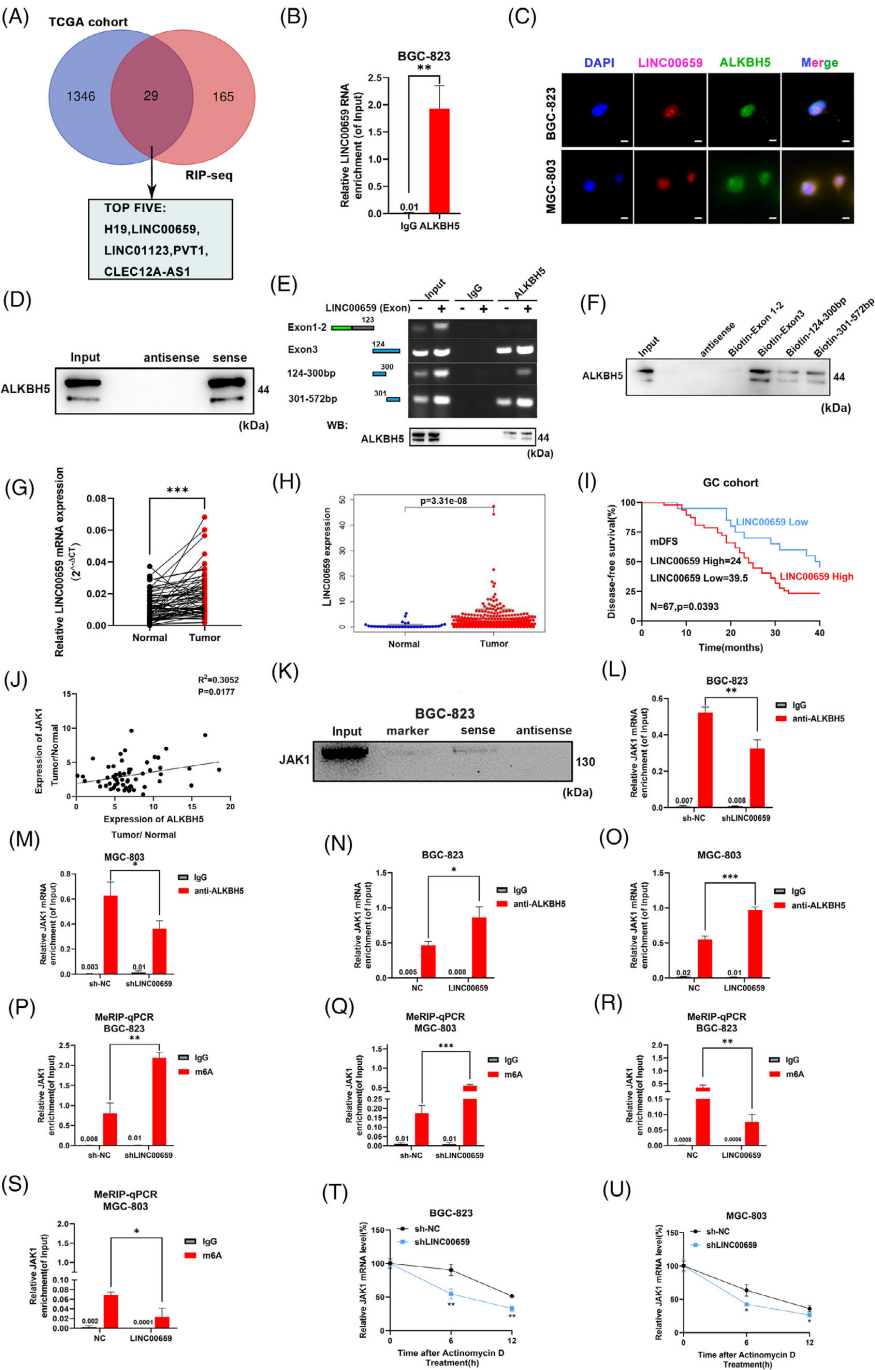
LINC00659 facilitates alkB homologue 5 (ALKBH5) binding to JAK1 mRNA. (A) The Venn diagram shows the lncRNAs detected by RNA binding protein immunoprecipitation sequencing (RIP‐seq) and The Cancer Genome Atlas (TCGA) database; top five candidate lncRNAs are shown. (B) RIP‐qPCR confirmed LINC00659 binding to ALKBH5. (C) RNA–protein colocalisation of LINC00659 with ALKBH5 in BGC‐823 and MGC‐803 cells (scale bars = 25 µm). (D) RNA pull‐down assay confirmed ALKBH5 binding to LINC00659. The RNA and protein in the RNA–protein complex were then detected by western blotting. (E) RIP and western blotting assays using ALKBH5 antibody revealing the interaction between LINC00659 and ALKBH5 protein in BGC‐823 cells transfected with a series truncated form of LINC00659. The immunoglobulin G (IgG)‐bound RNA was taken as a negative control. (F) RNA pull‐down assay demonstrating the interaction between LINC00659 truncations and ALKBH5 protein in BGC‐823 cells. (G) The levels of LINC00659 expression in gastric cancer (GC) and matched adjacent gastric tissues were detected by quantitative real‐time polymerase chain reaction (qRT‐PCR) (*n* = 67). (H) LINC00659 expression level was analysed in GC and normal tissues in TCGA database. (I) Kaplan–Meier analysis revealed DFS in GC patients based on the relative LINC00659 expression (*n* = 67). (J) LINC00659 expression was positively correlated with JAK1 expression in GC cohort (linear regression). (K) RNA pulldown assays confirmed the physical interaction between JAK1 and LINC00659 in BGC‐823 cells. (L‐O) RIP‐qPCR assays confirmed that overexpressing LINC00659 could enhance the binding between ALKBH5 and JAK1, while suppressing LINC00659 could inhibit the binding in BGC‐823 and MGC‐803 cells. (P‐S) MeRIP‐qPCR analysis was performed to detect the LINC00659‐mediated m^6^A modifications on JAK1. The m^6^A modifications on JAK1 were enhanced when LINC00659 knockdown while decreased when LINC00659 overexpression in BGC‐823 and MGC‐803 cells. (T‐U) LINC00659 on mRNA stability of JAK1 was detected by use of actinomycin D (2 µg/mL) on BGC‐823 and MGC‐803 cells with stable LINC00659 knockdown. ^*^ *p* < .05; ^**^ *p* < .01; ^***^ *p* < .001.

The lncRNA FOXM1‐AS promotes the interaction between ALKBH5 and FOXM1, and ALKBH5 decreases the m^6^A modification of FOXM1,^15^ which suggests that specific lncRNAs are involved in the interaction between the demethylase ALKBH5 and mRNA. Based on this hypothesis, we examined whether specific lncRNAs helped ALKBH5 remove the m^6^A modification on JAK1 mRNA. RIP sequencing (RIP‐seq) in BGC‐823 cells showed 736 combination peaks from 194 lncRNAs that were enriched with an anti‐ALKBH5 antibody (FDR <0.05). Analysis of the TCGA database found that 1375 lncRNAs were upregulated in GC tissues. There were 29 lncRNAs that overlapped between the RIP‐seq results and TCGA database. We selected the top five lncRNAs according to the count number in RIP‐seq data (Figure 5A). We silenced these five lncRNAs in BGC‐823 cells and found that only the silencing of LINC00659 caused the downregulation of JAK1 mRNA, which suggested that LINC00659 regulated JAK1 (Figure S5a).

The silencing of LINC00659 also downregulated the protein expression of JAK1, and the overexpression of LINC00659 caused JAK1 upregulation (Figure S5b,c). These results indicated that LINC00659 promoted JAK1 expression.

Similar to the RIP‐seq results, RIP, colocalisation and pull‐down assays confirmed that ALKBH5 and LINC00659 directly interacted with each other (Figure 5B‐D). However, LINC00659 did not influence the mRNA or protein level of ALKBH5, and vice versa (Figure S5d–g). RIP‐seq data showed that LINC00659 transcript 2 was enriched with anti‐ALKBH5 antibody, which has three exons (exon 1: 1–51 bp; exon 2: 52–123 bp; exon 3: 124–572 bp). To investigate the specific region on LINC00659 where ALKBH5 bound, we divided LINC00659 into four parts (1–123 bp; 124–300 bp; 301–572 bp; 124–572 bp). The RIP and pull‐down assays showed that ALKBH5 primarily bound to exon 3 of LINC00659 (124–572 bp), which was mostly located at 301–572 bp (Figure 5E,F). To further investigate the specific regions of ALKBH5 that were responsible for LINC00659 binding, we performed RIP assays. Region 3 was mostly responsible for LINC00659 binding (Figure S5h,i).

To verify whether LINC00659 was upregulated in GC samples, RT‐qPCR assay was used to detect the expression level of LINC00659 in 67 pairs of GC and adjacent normal tissues. We found that LINC00659 had a high level of expression in GC tissues (Figure 5G). These results were consistent with the TCGA dataset, which indicated the overexpression of LINC00659 in GC tissues (Figure 5H).We separated 67 patients into LINC00659‐High and LINC00659‐Low groups according to the expression level of LINC00659 in GC samples. Kaplan–Meier survival curves revealed that high LINC00659 expression group had a shorter DFS than group with low LINC00659 expression (Figure 5I). Gene expression analysis demonstrated a positive correlation between LINC00659 and JAK1 in our cohort and the TCGA database (Figures 5J and S6a). The higher expression of LINC00659 in BGC‐823 and MGC‐803 was also observed by using RT‐qPCR (Figure S6b). Fluorescence in situ hybridisation and nuclear‐cytoplasmic fractionation analysis suggested that LINC00659 was primarily enriched nucleus in cell nucleus (Figure S6c–e).

Based on the positive correlation between upregulated LINC00659 in GC and JAK1 expression and the direct binding of LINC00659 to ALKBH5, we hypothesised that LINC00659 facilitated the ALKBH5–JAK1 interaction. The direct interaction between JAK1 and LINC00659 was confirmed by pull‐down assays (Figure 5K). We established ALKBH5 knockout BGC‐823 and MGC‐803 cells using the Cas9 gene editing system. The mRNA and protein levels of JAK1 in GC cells were reduced following knockout of ALKBH5. However, ALKBH5 knockout and LINC00659 overexpression simultaneously did not return these levels to normal (Figure S6f–h). These results indicated that LINC00659‐promoted JAK1 expression relied on ALKBH5.

A series of RIP assays revealed that less JAK1 mRNA was enriched with an ALKBH5 antibody after silencing of LINC00659, and overexpressing LINC00659 resulted in an increased combination between ALKBH5 and JAK1 transcripts (Figure 5L‐O). The abundance of m^6^A on JAK1 mRNA was downregulated without LNC00659, and the opposite result was observed when LINC00659 was overexpressed (Figure 5P‐S). JAK1 mRNA was more stable in ActD‐treated BGC‐823 and MGC‐803 cells overexpressing LINC00659, and JAK1 mRNA exhibited a faster degradation rate when LINC00659 was silenced in ActD‐treated BGC‐823 and MGC‐803 cells (Figures 5T‐U and S6i,j).

In general, LINC00659 helped ALKBH5 bind to JAK1 mRNA, which removed m^6^A on JAK1 mRNA and enhanced its stability to cause the upregulation of JAK1.

### 3.6 LINC00659 promotes GC proliferation and metastasis in vitro and in vivo

To verify the biological function of LINC00659 in GC progression, we stably knocked down and overexpressed LINC00659 in BGC‐823 and MGC‐803 cells for further function assays (Figure S7a,b). CCK8 and EdU assays showed that knockdown of LINC00659 prominently inhibited GC cell growth in vitro (Figure S7c,e). Similar results were detected in colony formation assays (Figure S7g). However, the opposite results were observed when LINC00659 was overexpressed (Figure S7d,f,h). The suppression of LINC00659 attenuated the in vitro metastasis and invasion of GC cells as indicated by Transwell assays (Figure S7i), and the overexpression of LINC00659 promoted the invasive and migratory capacities of GC cells (Figure S7j).

To investigate the potential roles of LINC00659 in GC cells in vivo, LINC00659‐deficient and LINC00659‐overexpressing BGC‐823 cells were used to establish nude mouse xenograft model. Compared to the control group, the weight and volume of tumours from the sh‐LINC00659 group were much smaller (Figure S8a–c). RT‐qPCR assays verified the knockdown of LINC00659 expression in mouse tumour tissues (Figure S8d). IHC staining also showed lower Ki‐67 signals in the LINC00659‐knockdown group (Figure S8e). The tumours harvested from the LINC00659‐overexpressing group were larger and heavier than tumours from the NC group (Figure S8h–j), with higher Ki‐67 protein levels (Figure S8l). The expression of LINC00659 in tumour tissues was evaluated using RT‐qPCR (Figure S8k).

We also injected the abovementioned cells into nude mice via the tail vein to investigate in vivo metastasis ability. Fewer metastatic nodes were detected in the lung in the LINC00659‐silenced group (Figure S8f, g), and more nodes were detected in the lung and liver in the LINC00659‐overexpressing group (Figure S8m–o) than in the NC group.

In conclusion, LINC00659 increased proliferating and metastatic GC cells.

### 3.7 LINC00659 facilitates ALKBH5 to promote the GC progression by upregulating JAK1

**FIGURE 6:**
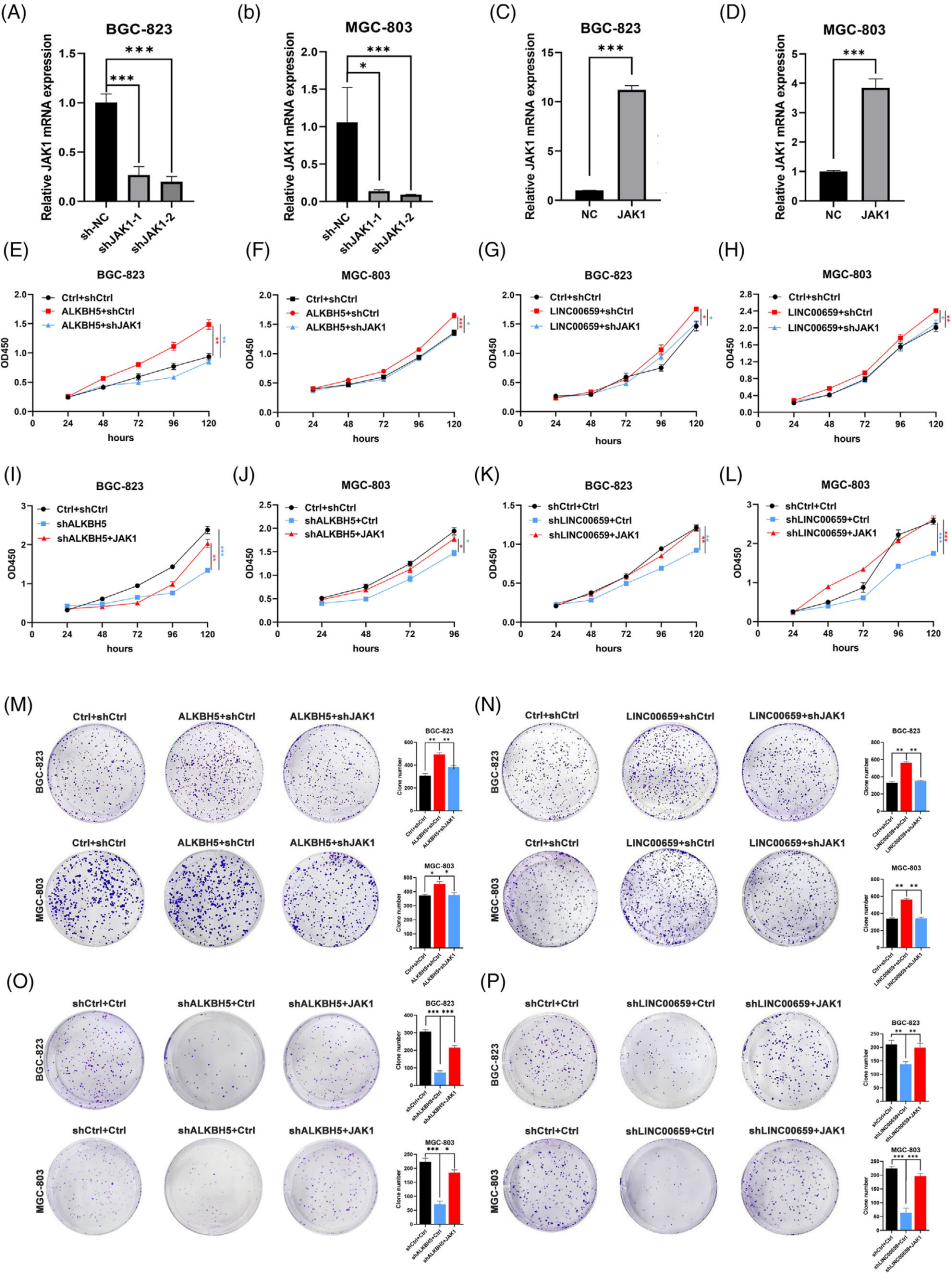
LINC00659 facilitates alkB homologue 5 (ALKBH5) to promote the gastric cancer (GC) progression by upregulating JAK1. (A‐D) The mRNA levels of JAK1 in GC cells with JAK1 knockdown or overexpression were detected by quantitative real‐time polymerase chain reaction (qRT‐PCR). (E‐H) cell counting kit‐8 (CCK8) assays were conducted to determine cell proliferation of ALKBH5/LINC00659‐overexpressing BGC‐823 and MGC‐803 cells transfected with the JAK1 shRNAs or their corresponding controls. (I‐L) CCK8 assays were conducted to determine cell proliferation of ALKBH5/LINC00659‐silencing BGC‐823 and MGC‐803 cells transfected with the JAK1‐overexpressing plasmids or their corresponding controls. (M and N) Colony‐formation assays were conducted to determine the colony‐formation ability of ALKBH5/LINC00659‐overexpressing GC cells transfected with the JAK1 shRNAs or their corresponding controls. (O and P) Colony‐formation assays were conducted to determine the colony‐formation ability of ALKBH5/LINC00659‐silencing GC cells transfected with the JAK1‐overexpressing plasmids or their corresponding controls. ^*^ *p* < .05; ^**^ *p* < .01; ^***^ *p* < .001.

To further examine whether LINC00659 and ALKBH5 affected the abilities of GC cells proliferation and invasion by modulating JAK1 expression, we performed following plenty of rescue experiments. We designed shRNAs to target JAK1 and confirmed the knockdown efficiency using RT‐qPCR (Figure 6A,B). Silencing JAK1 suppressed cell proliferation induced by ALKBH5 and LINC00659 (Figures 6E‐H,M‐N and S9a–d), and cell invasion and migration (Figure S10a–d). When JAK1 was overexpressed (Figure 6C,D),ALKBH5/LINC00659 knockdown inhibited GC cell proliferation, migration and invasion (Figures 6I‐L,O‐P, S9e–h and S10e–h). To validate the mechanism of LINC00659 facilitation of ALKBH5 upregulation of JAK1 mRNA expression, we designed a series of functional experiments. Silencing LINC00659 suppressed ALKBH5‐induced cell proliferation in vitro and cell invasion and migration (Figure S11a–c). The silencing of LINC00659 or JAK1 impaired the ALKBH5 overexpression‐induced increase in GC cell proliferation in vivo (Figure S11d–f). Sections of tumour xenografts from ALKBH5‐overexpressing or ALKBH5‐overexpressing BGC‐823 cells with LINC00659/JAK1 knockdown subcutaneously injected into nude mice were stained with JAK1 antibodies. Silencing LINC00659 or JAK1 appreciably supressed the increase of JAK1 staining induced by ALKBH5 (Figure S11g). In conclusion, LINC00659 facilitated ALKBH5 to promote the growth and invasion of GC cells in vivo and in vitro by upregulating JAK1.

### 3.8 JAK1 upregulation activates the JAK1/STAT3 pathway in GC

**FIGURE 7:**
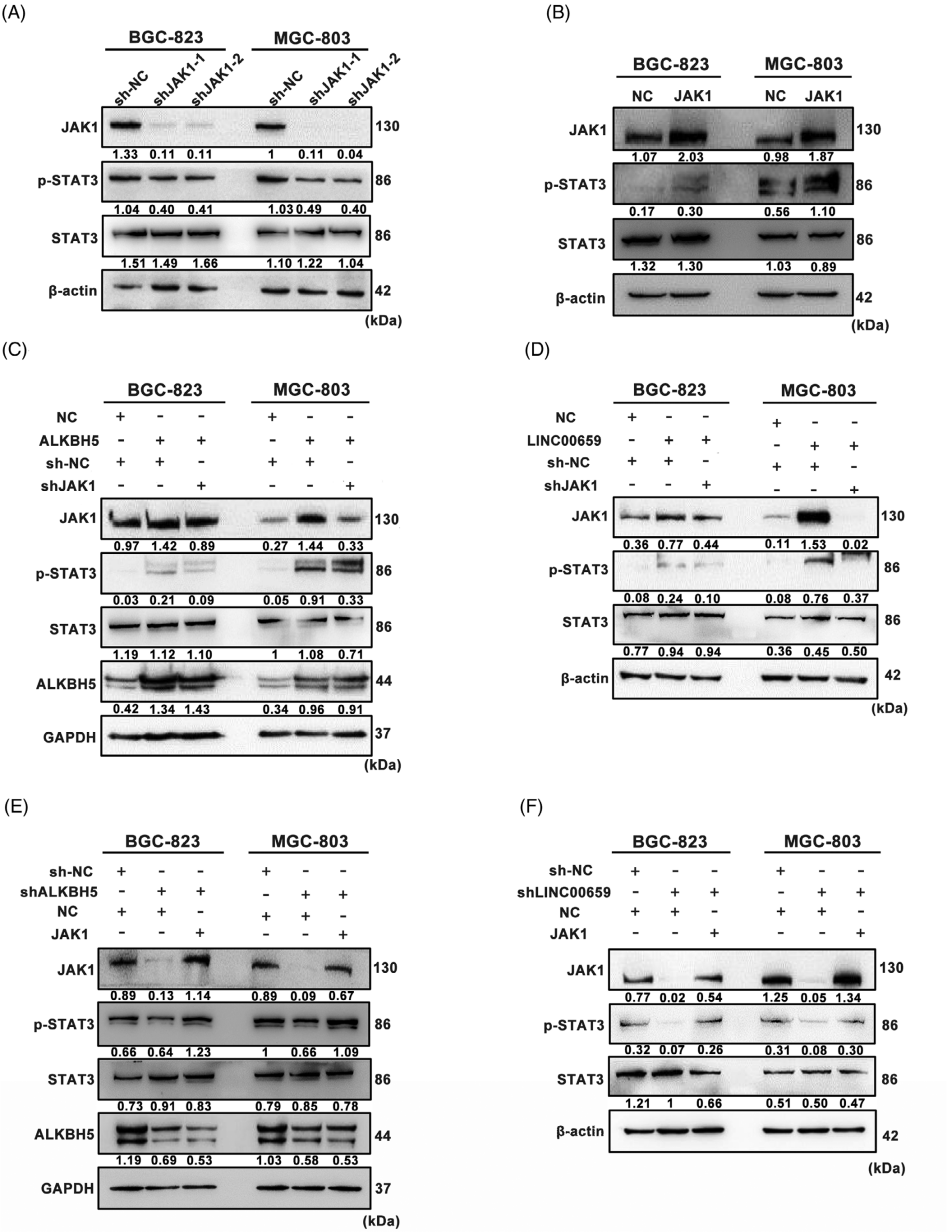
JAK1 upregulation activates JAK1/STAT3 pathway in gastric cancer (GC). (A and B) The protein levels of JAK1, p‐STAT3 and STAT3 were detected by western blotting in GC cells transfected with the JAK1 shRNAs (A) or JAK1‐overexpressing plasmids (B). Relative protein levels were normalised to β‐actin. (C and D) The protein levels of JAK1, p‐STAT3 and STAT3 in alkB homologue 5 (ALKBH5)/LINC00659‐overexpressing GC cells transfected with the JAK1 shRNAs or their corresponding controls were detected by western blotting. Relative protein levels were normalised to β‐actin or GAPDH. (E and F) The protein levels of JAK1, p‐STAT3 and STAT3 in ALKBH5/LINC00659‐silencing GC cells transfected with the JAK1‐overexpressing plasmids or their corresponding controls were detected by western blotting. Relative protein levels were normalised to β‐actin or GAPDH.

JAK1 is one of the JAK that phosphorylates proteins of the STAT family and plays a crucial role in multiple cancers, including GC. Previous researches have reported that the JAK1/STAT3 pathway activation promoted GC cell proliferation, invasion and metastasis.^24^ To verify the target of JAK1, we detected the phosphorylation of STAT1, STAT3 and STAT5 after JAK1 silencing (shJAK1‐1, shJAK1‐2) in GC cells using WB and found a significant decrease in phosphorylated STAT3 but not STAT1 or STAT5 (Figure S12a,b). Therefore, we chose STAT3 as the substrate of JAK1 in GC cells. In contrast to the phenomenon that silencing JAK1 caused the downregulation of phosphorylated STAT3 (p‐STAT3) (Figure 7A), the phosphorylation of STAT3 was dramatically increased after overexpressing JAK1 in GC cells (Figure 7B). To further examine the role of ALKBH5 and LINC00659 in activating the JAK1/STAT3 pathway, we performed rescue experiments. WB demonstrated that ALKBH5/LINC00659 overexpression upregulated the expression of JAK1 and p‐STAT3, and suppression of JAK1 abrogated the effect of ALKBH5/LINC00659 on JAK1 upregulation and STAT3 activation (Figure 7C,D). In contrast, silencing ALKBH5/LINC00659 downregulated the expression of JAK1 and p‐STAT3, and overexpressing JAK1 rescued the suppression of JAK1 and p‐STAT3 caused by the knockdown of ALKBH5/LINC00659 (Figure 7E,F). In summary, JAK1, upregulated by ALKBH5 and LINC00659, phosphorylated STAT3 and activated the JAK1/STAT3 pathway, which accelerated the progression of GC.

### 3.9 Clinical correlation between ALKBH5/LINC00659 and JAK1 in GC

For further investigation of the relationship between ALKBH5/LINC00659 and JAK1 in the GC, we validated the JAK1 expression in tumours harvested from nude mice with LINC00659/ALKBH5 abnormally expressed and the paired controlled ones. IHC staining assays demonstrated that knocking down LINC00659/ALKBH5 decreased JAK1 expression in tumours, while overexpressing LINC00659/ALKBH5 increased the expression of JAK1 (Figure S13a).

To further investigate the clinical relationship between ALKBH5/LINC00659 and JAK1 in GC, we examined JAK1 and ALKBH5 expression in neoplastic tissues from patients with GC and divided tissues into ALKBH5‐Low and ALKBH5‐High groups according to ALKBH5 expression. The expression of ALKBH5 in GC positively correlated with the expression of JAK1 (Figure S13b,c). In summary, the ALKBH5–LINC00659/m^6^A/JAK1 axis stimulated the development of GC.

## 4 DISCUSSION

The m^6^A modification of mRNAs and noncoding RNA was the most common epigenetic modification among eukaryotic cells.^31^ Increasing evidence indicates that m^6^A modifications are associated with tumourigenesis, metastasis and angiogenesis of gastrointestinal tumours.^32^, ^33^, ^34^ Aberrant expression of m^6^A‐associated enzymes, such as m^6^A writers (the METTL3/METTL14/WTAP complex) and readers (YTHDF1, YTHDF2), often leads to GC progression.^35^, ^36^, ^37^, ^38^, ^39^

Recent studies have revealed that the m^6^A demethyltransferase ALKBH5 acted as a double‐edged sword in tumourigenesis. ALKBH5 promoted cancer stem cell self‐renewal in acute myeloid leukaemia,^40^ and caused glioblastoma tumourigenesis by sustaining FOXM1 expression.^15^ On the other hand, ALKBH5 inhibited pancreatic cancer progression by activating PER1.^41^ However, research on ALKBH5 in GC progression is controversial. Zhang et al.^20^ reported that ALKBH5 demethylated the lncRNA NEAT1 and promoted GC invasion and metastasis. ALKBH5 bound to NEAT1 and influenced the expression of EZH2 (a subunit of the polycomb repressive complex). Hu et al.^21^ reported that ALKBH5 was a cancer suppressor in GC. ALKBH5 suppressed the invasion of GC by downregulating and removing the m^6^A modifications of PKMYT1, which enhanced the invasion of GC. These controversial results reveal that the role of ALKBH5 in GC progression must be fully elucidated. Based on three bioinformatics methods, we analysed the entire TCGA database and found significant upregulation of ALKBH5 in 414 GC tumour samples compared to 37 normal samples. Comparing GC tissues with paired noncancerous tissues (*n* = 67), we discovered a significant increase in ALKBH5 expression. However, Hu et al. analysed only 290 GC tumour samples and 22 normal samples obtained from the TCGA‐Stomach adenocarcinoma database, and the number of patients included in our GC cohort was 49.^21^ We analysed the ACRG GC cohort (*n* = 300) and found a shorter OS, RFS and DFS with higher ALKBH5 expression in GC.^42^ These data were more convincing because of the larger sample size, high integrity of information and high‐quality clinical follow‐up. By analysing clinicopathological characteristics, there was an association between ALKBH5 and histological differentiation, invasion depth, TNM stage and lymphatic metastasis. We also revealed that high ALKBH5 expression led to poor prognosis in GC patients as shown by Kaplan–Meier survival analysis. We confirmed that ALKBH5 functioned as an oncogenic molecule and accelerated GC proliferation, metastasis and invasion using in vivo and in vitro experiments in ALKBH5 stably silenced/overexpressed BGC‐823 and MGC‐803 cells. To make our study more convincing, we repeated cell proliferation and invasion experiments in AGS and NCI‐N87 cells, and similar results were observed.

Using MeRIP‐seq and RNA‐seq, we found that JAK1 may be the downstream target of ALKBH5 in GC. JAK1 is a member of the JAK family and phosphorylates STAT proteins. After moving from the cytoplasm to the nucleus, phosphorylated STATs activate genes involved in cell survival and proliferation.^22^ Activated JAK1/STAT3 plays a crucial role in GC proliferation and metastasis.^24^ We showed that JAK1 was overexpressed in GC tissues and associated with poor outcome. ALKBH5 bound to JAK1 and removed the m^6^A modification of JAK1 mRNA and then enhanced the stability of JAK1 mRNA. m^6^A reading proteins recognise target genes using m^6^A modification and play a role in RNA decay. A previous study noted the interaction between JAK1 and YTHDF2.^31^ The expression of JAK1 increased significantly after suppression of YTHDF2 in our study. Overexpressing ALKBH5 weakened the binding between YTHDF2 and JAK1. These results demonstrated that the degradation of JAK1 mRNA was dependent on YTHDF2 and was reduced by ALKBH5, which upregulated the expression of JAK1.

Noncoding RNAs may function as scaffolds and facilitate the binding between mRNAs and proteins. We overlapped the RIP‐seq results and TCGA database and chose five target lncRNAs according to the count number in RIP‐seq data. After evaluating the regulatory effect of these five lncRNAs on JAK1, we found that LINC00659 directly bound to ALKBH5, with no effect on ALKBH5 expression. Because of LINC00659, ALKBH5 more easily bound to JAK1 mRNA and enhanced its stability. Therefore, we first confirmed that a long noncoding RNA, LINC00659, promoted the interaction of the ALKBH5–JAK1 complex and increased JAK1 mRNA expression in GC.

JAK1 is a key member of the JAK family, which participated in the JAK/STAT pathway. We found that STAT3, but not STAT1 or STAT5, was phosphorylated by JAK1 in GC cells. Upregulated JAK1 phosphorylated STAT3 and activated the JAK1/STAT3 pathway, which accelerated the progression of GC.

RNA methylation at m^6^A‐containing sites represents a new therapeutic target. For example, meclofenamic acid (MA), FTO‐IN‐5 and FTO‐IN‐4 are highly selective inhibitors of FTO. Huang et al.^43^ found that MA specifically competed with FTO binding to m^6^A‐containing sites and increased m^6^A levels in mRNA. However, ALKBH5‐targeting drugs need further exploration.

Therefore, our study provided compelling in vitro and in vivo evidence that LINC00659 and YTHDF2 function in the modification of ALKBH5‐mediated JAK1 mRNA to promote GC proliferation and metastasis. In addition to demonstrating a connection between ALKBH5 and long noncoding RNA, we further explored the pathways leading to GC development and occurrence and provided biological mechanisms behind GC development and future therapeutic opportunities.

## CONFLICT OF INTEREST STATEMENT

The authors declare no conflicts of interest.

## Supporting information

Supporting Information

Supporting Information

Supporting Information

Supporting Information

Supporting Information

Supporting Information

Supporting Information

Supporting Information

Supporting Information

Supporting Information

Supporting Information

Supporting Information

Supporting Information

Supporting Information

Supporting Information
