# Supplementary figures and images for: LINC00659 cooperated with ALKBH5 to accelerate gastric cancer progression by stabilising JAK1 mRNA in an m6A‐YTHDF2‐dependent manner
Source: Clin Transl Med. 2023 Mar 2;13(3):e1205. doi: 10.1002/ctm2.1205 (PMC9982078; doi:10.1002/ctm2.1205)

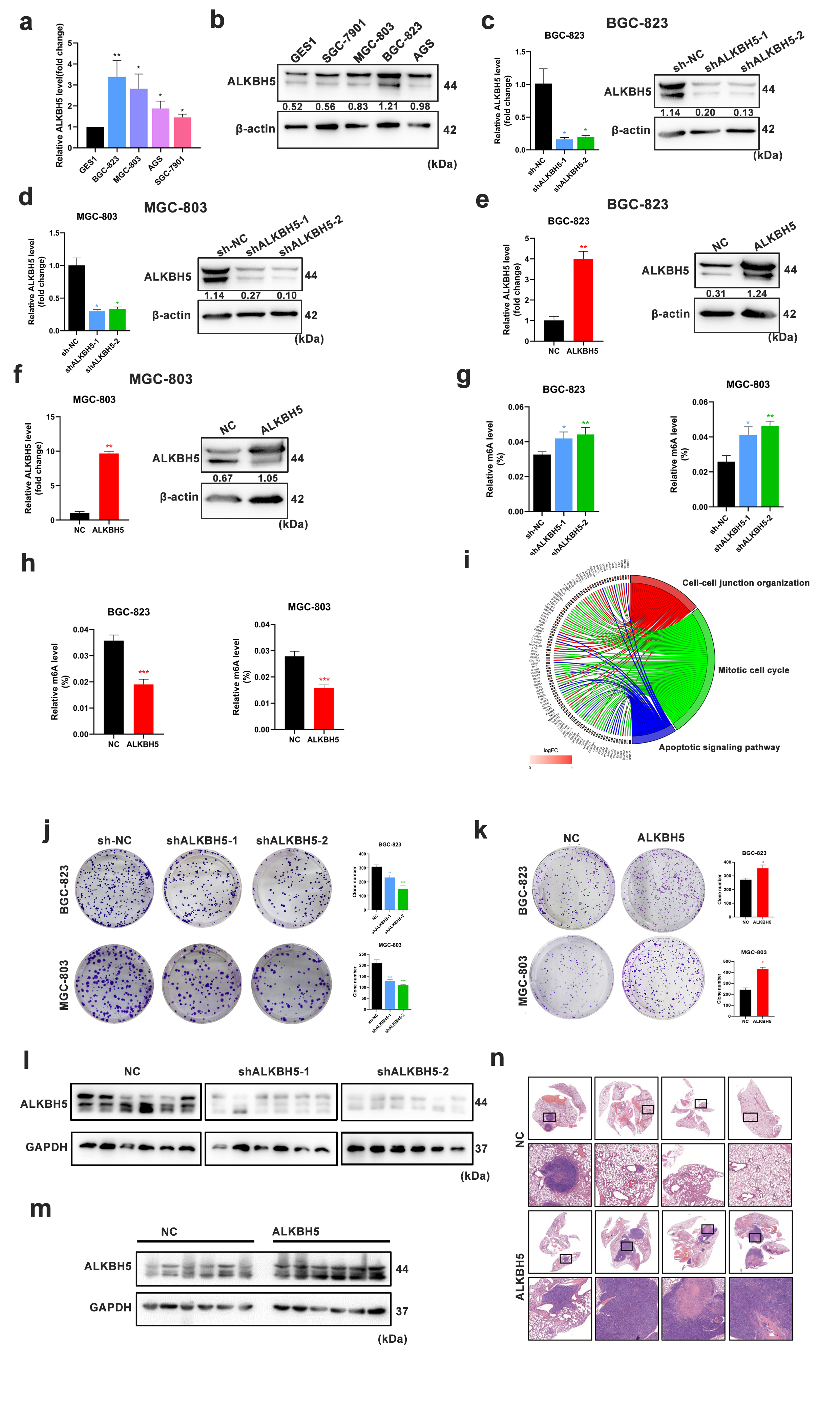

Supplement: Supplementary file 1 — Supporting Information [file CTM2-13-e1205-s005.tif]

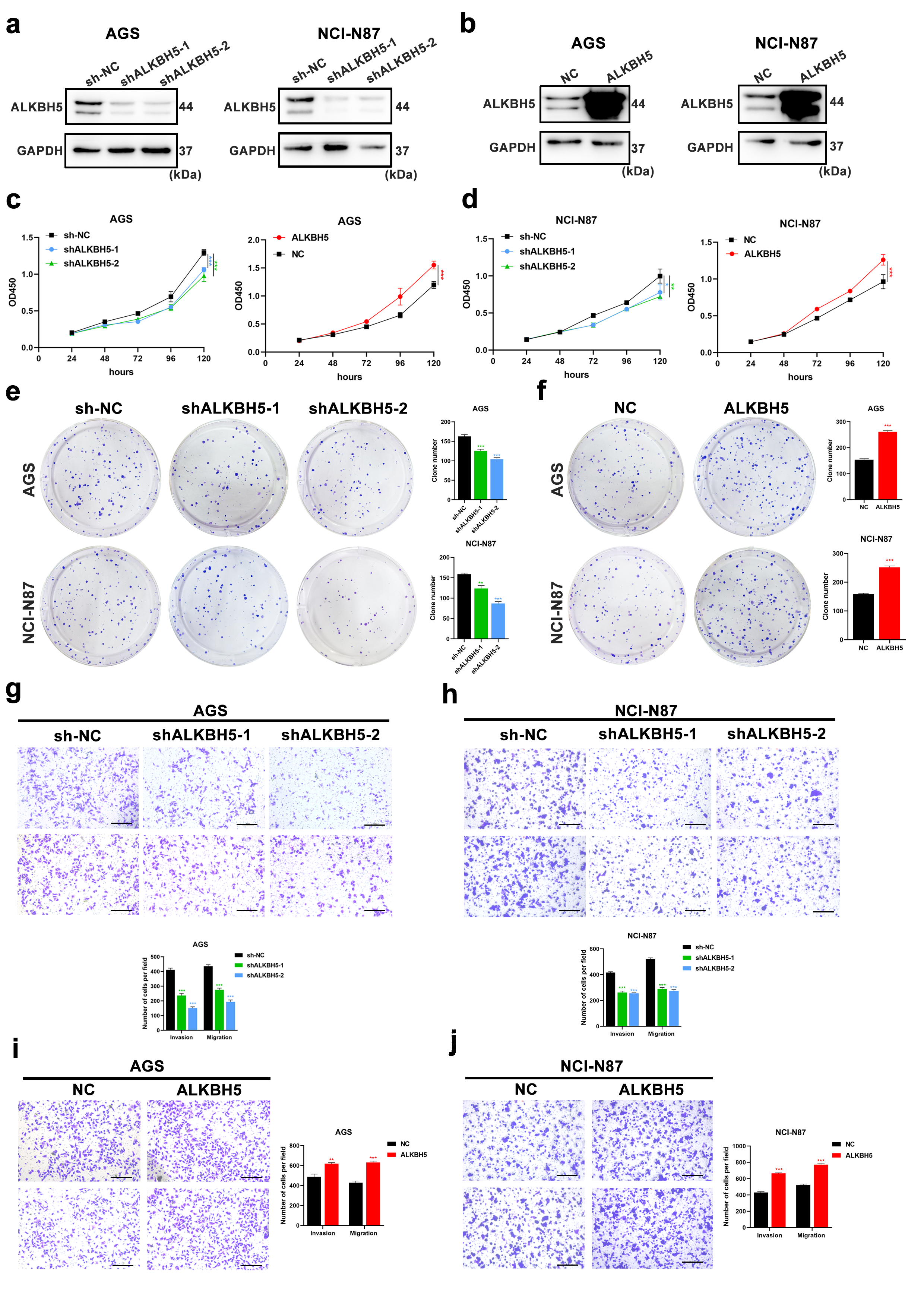

Supplement: Supplementary file 2 — Supporting Information [file CTM2-13-e1205-s003.tif]

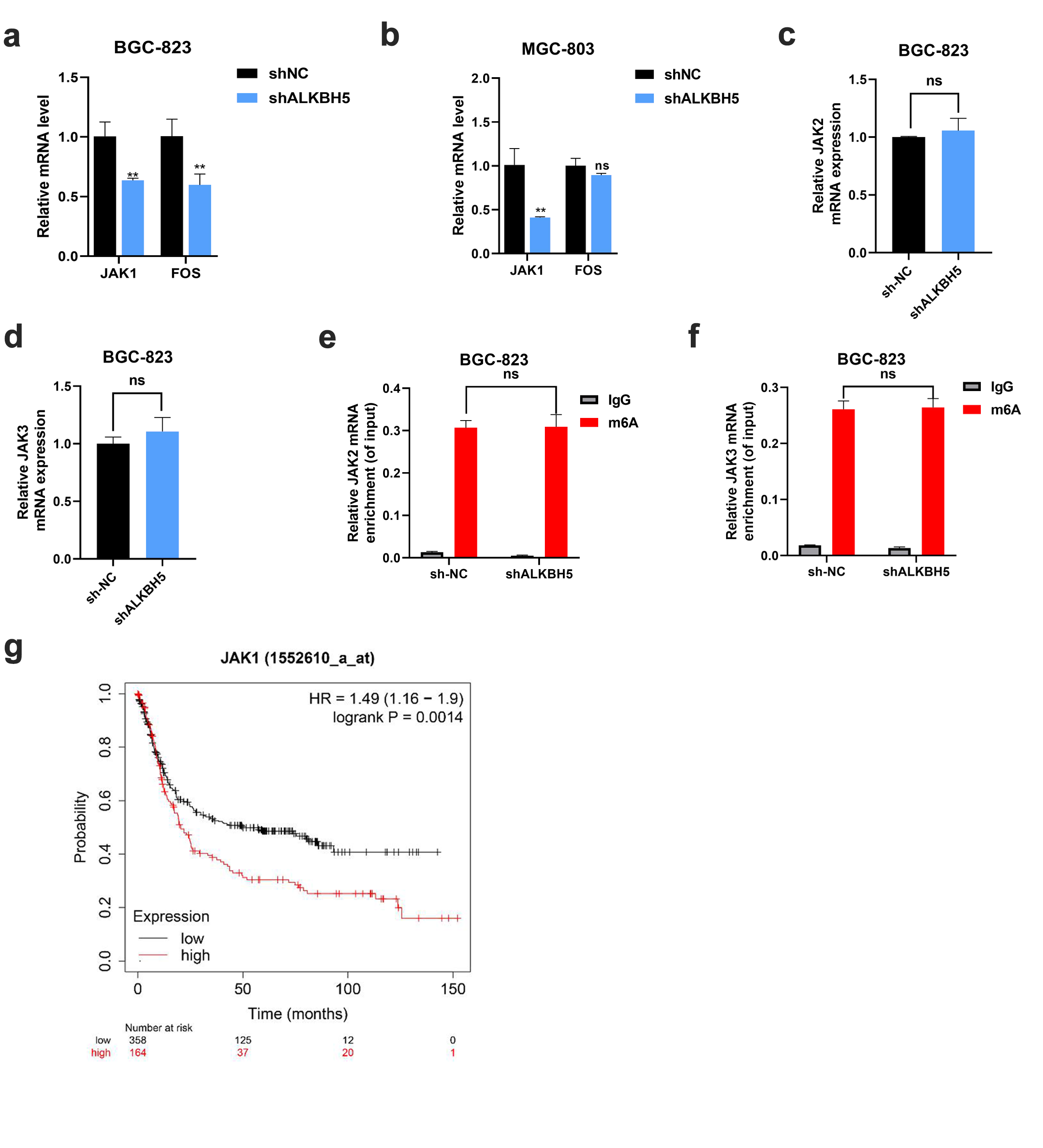

Supplement: Supplementary file 3 — Supporting Information [file CTM2-13-e1205-s011.tif]

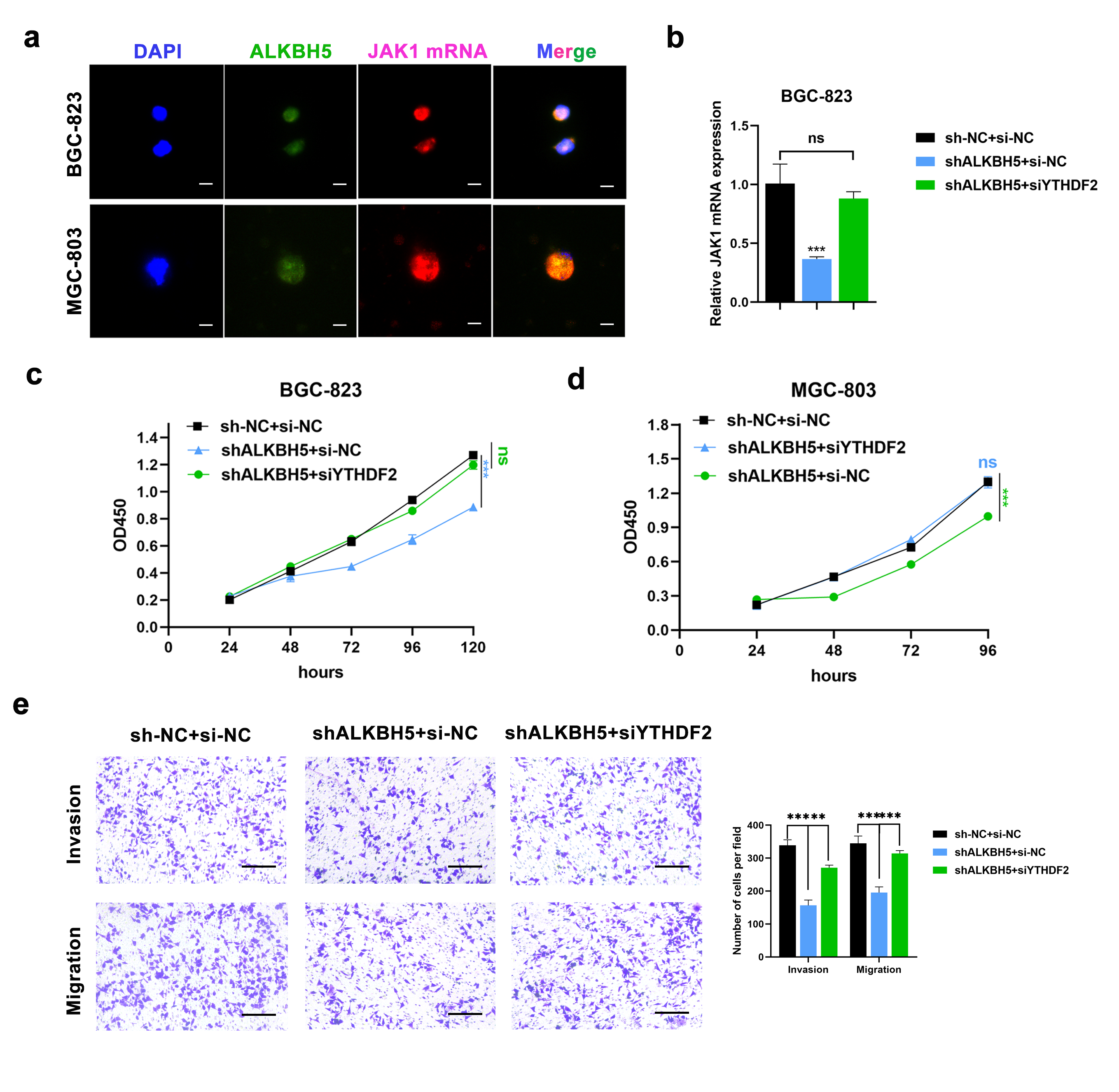

Supplement: Supplementary file 4 — Supporting Information [file CTM2-13-e1205-s015.tif]

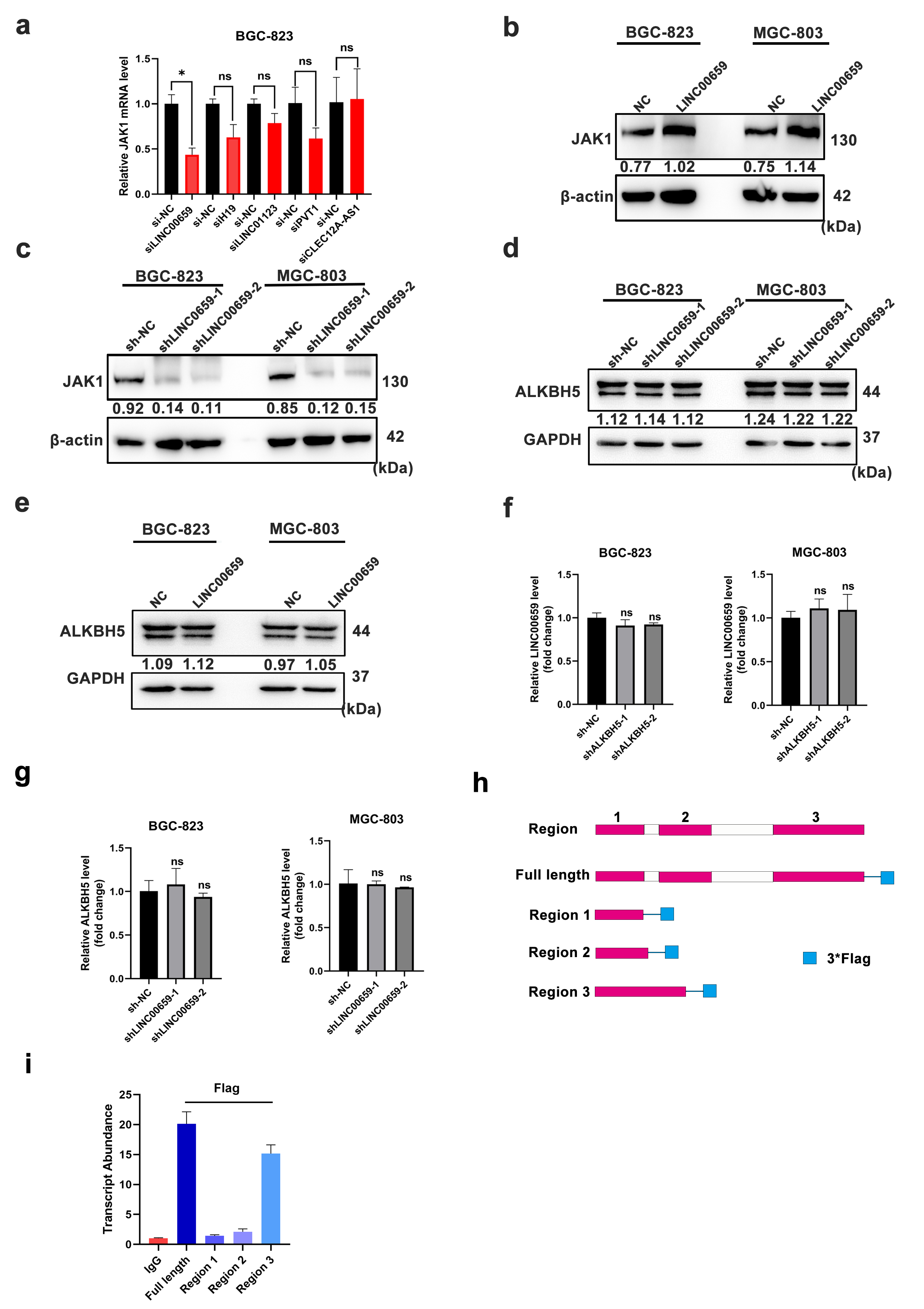

Supplement: Supplementary file 5 — Supporting Information [file CTM2-13-e1205-s010.tif]

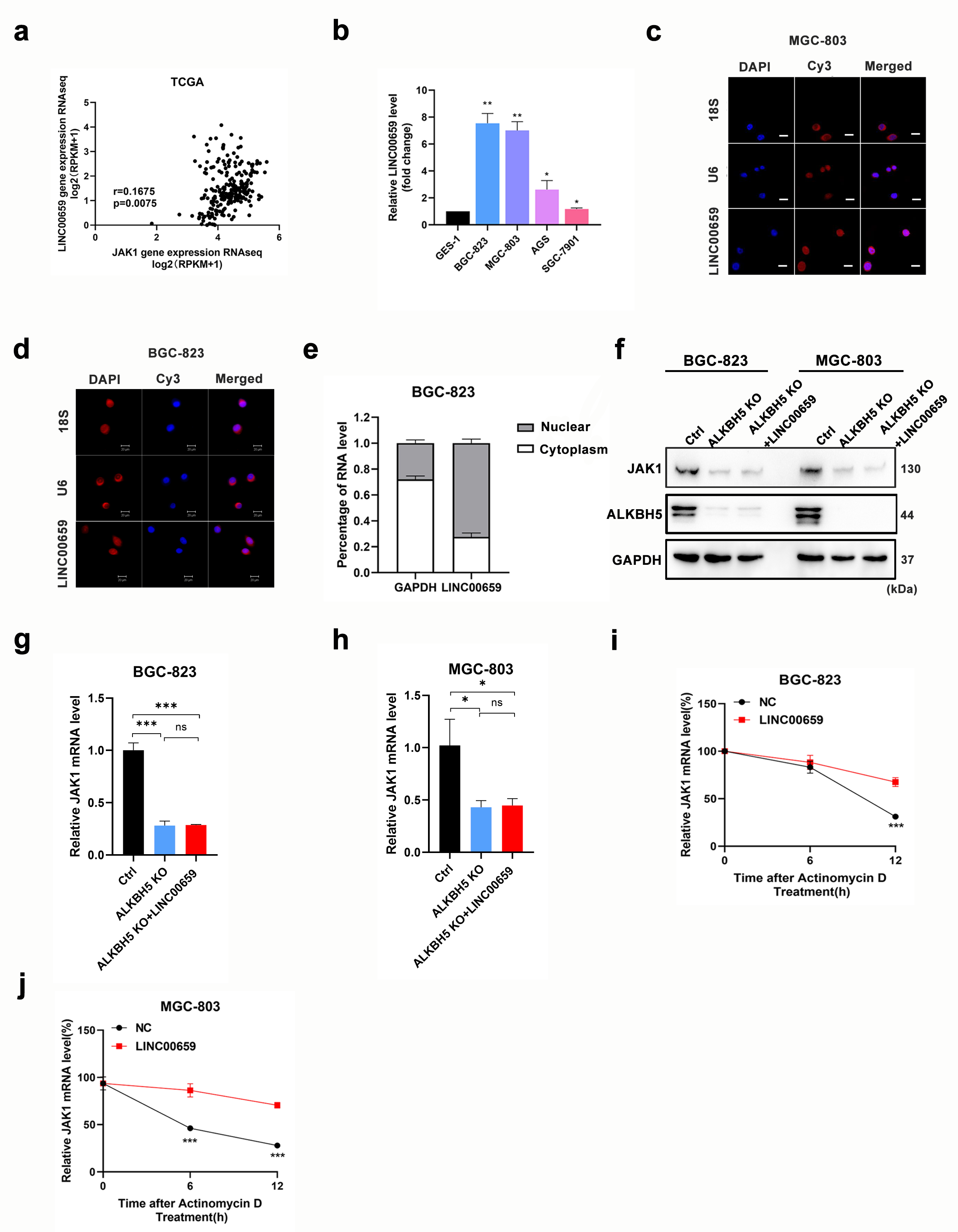

Supplement: Supplementary file 6 — Supporting Information [file CTM2-13-e1205-s009.tif]

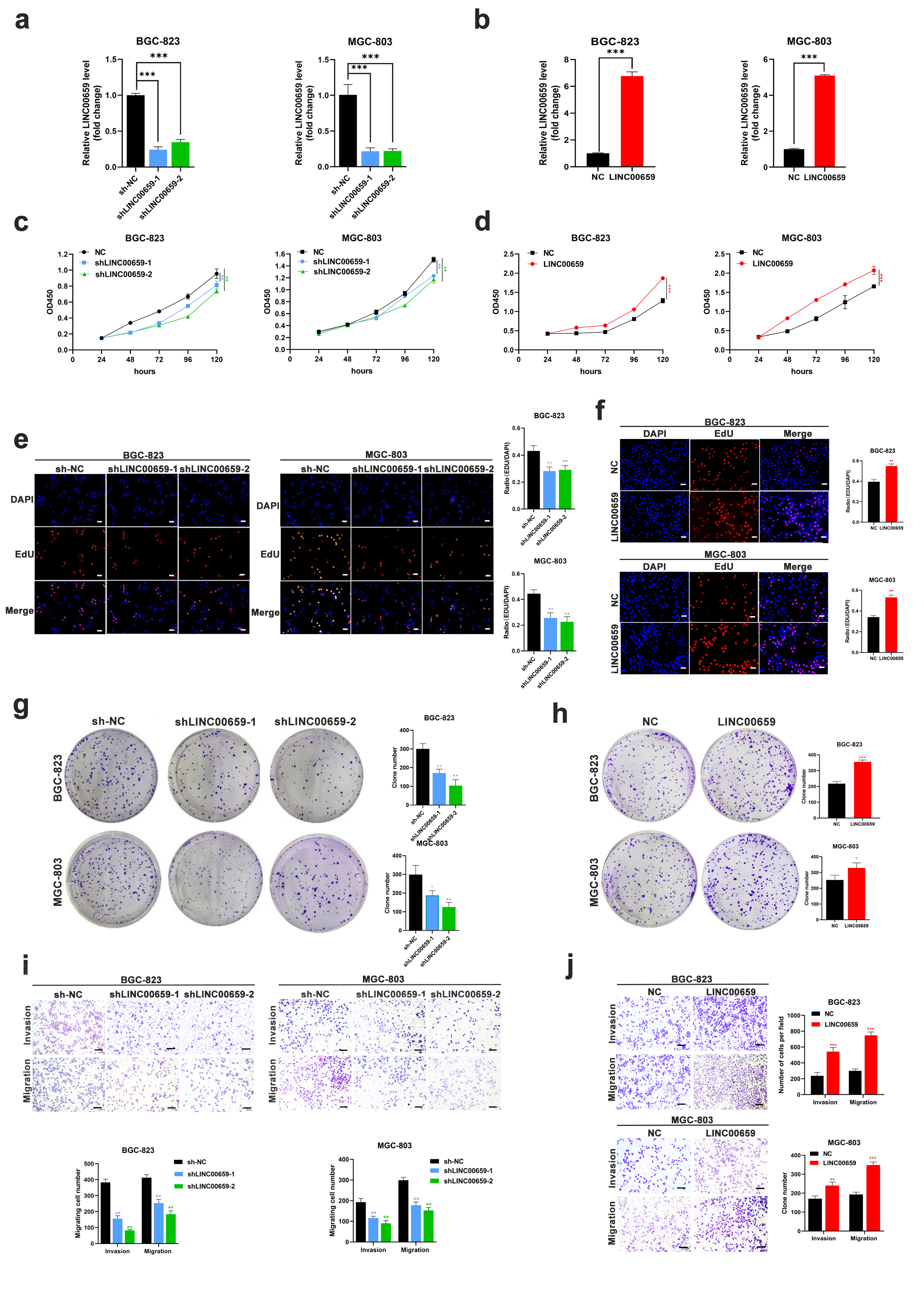

Supplement: Supplementary file 7 — Supporting Information [file CTM2-13-e1205-s012.tif]

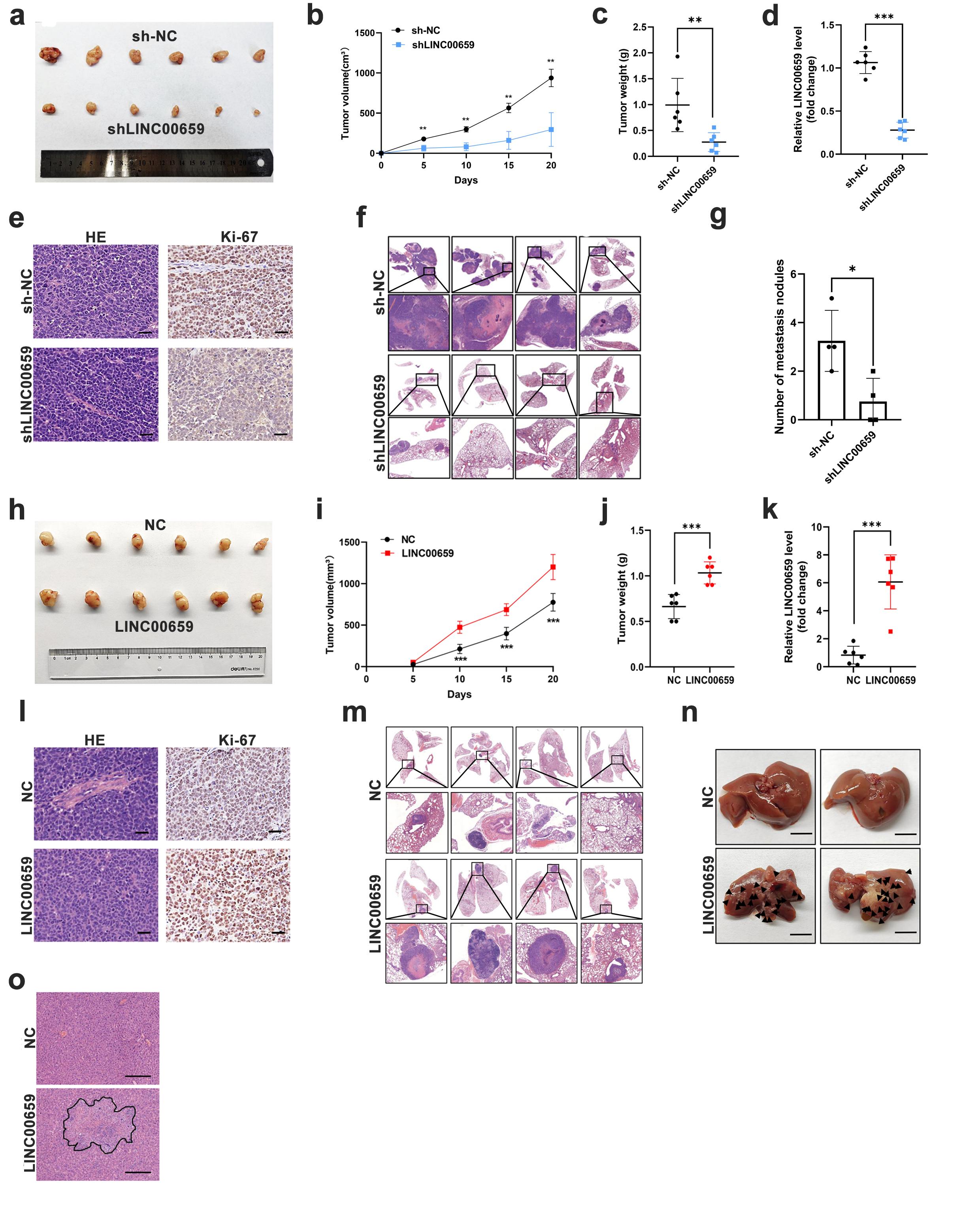

Supplement: Supplementary file 8 — Supporting Information [file CTM2-13-e1205-s001.tif]

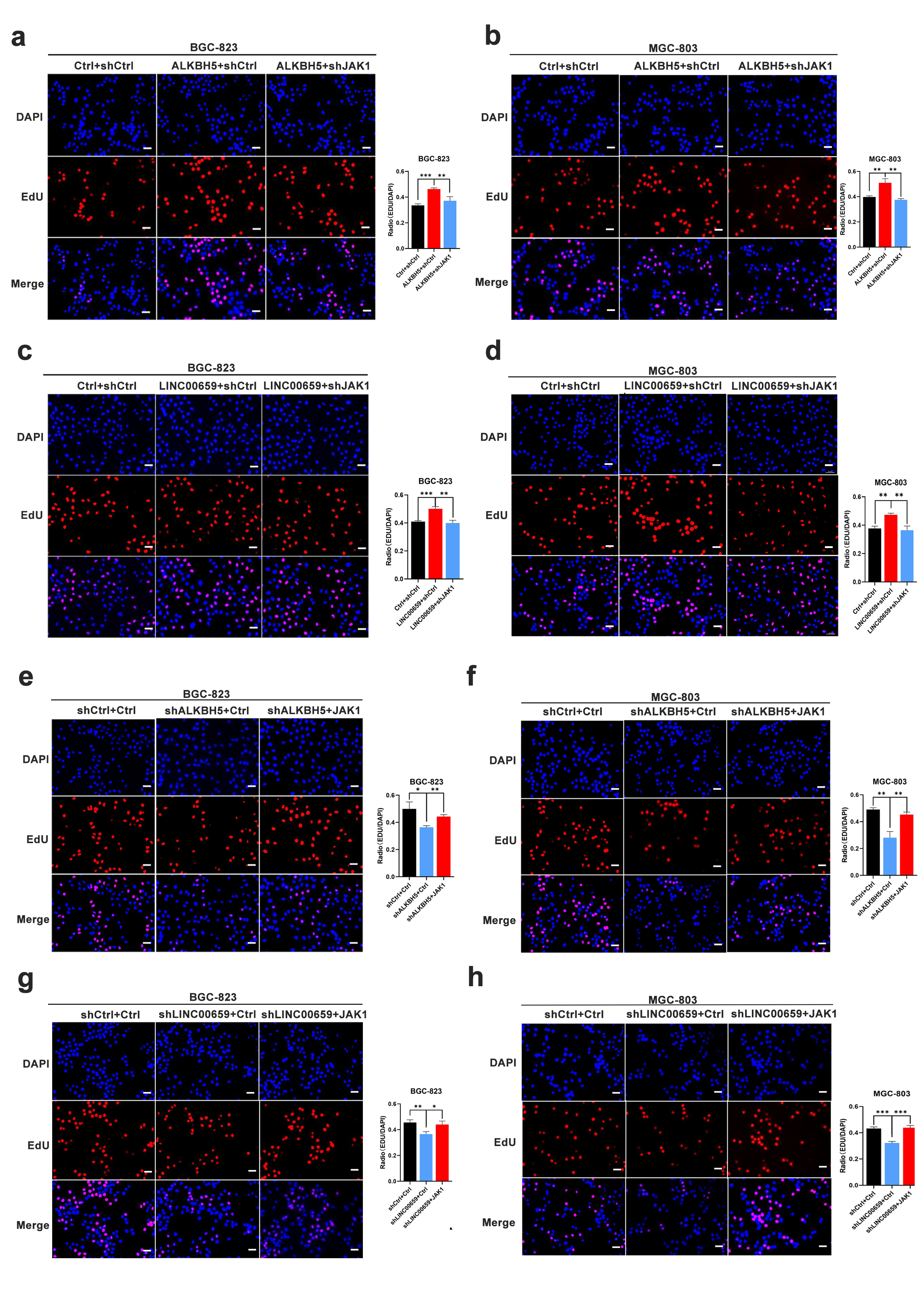

Supplement: Supplementary file 9 — Supporting Information [file CTM2-13-e1205-s013.tif]

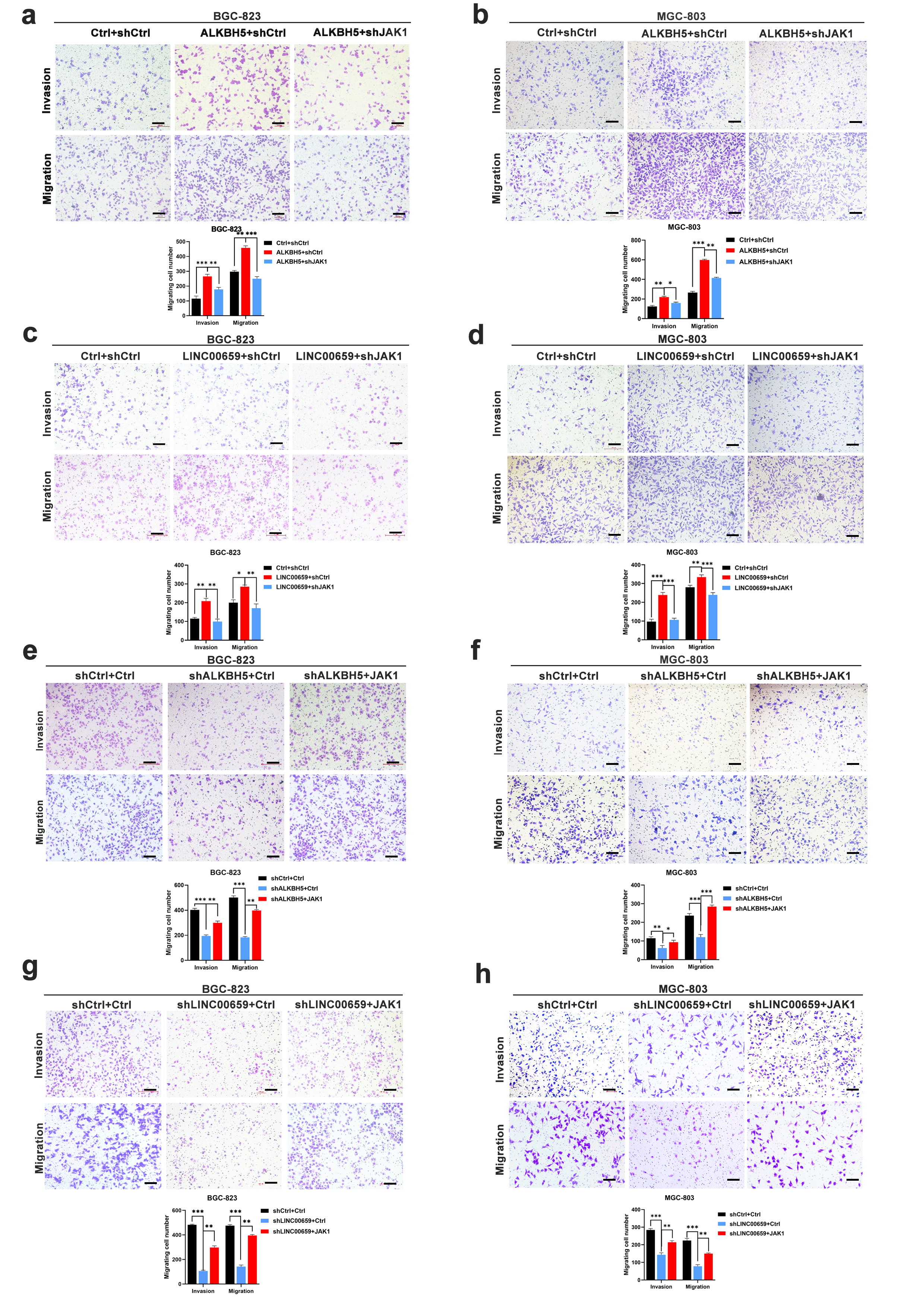

Supplement: Supplementary file 10 — Supporting Information [file CTM2-13-e1205-s008.tif]

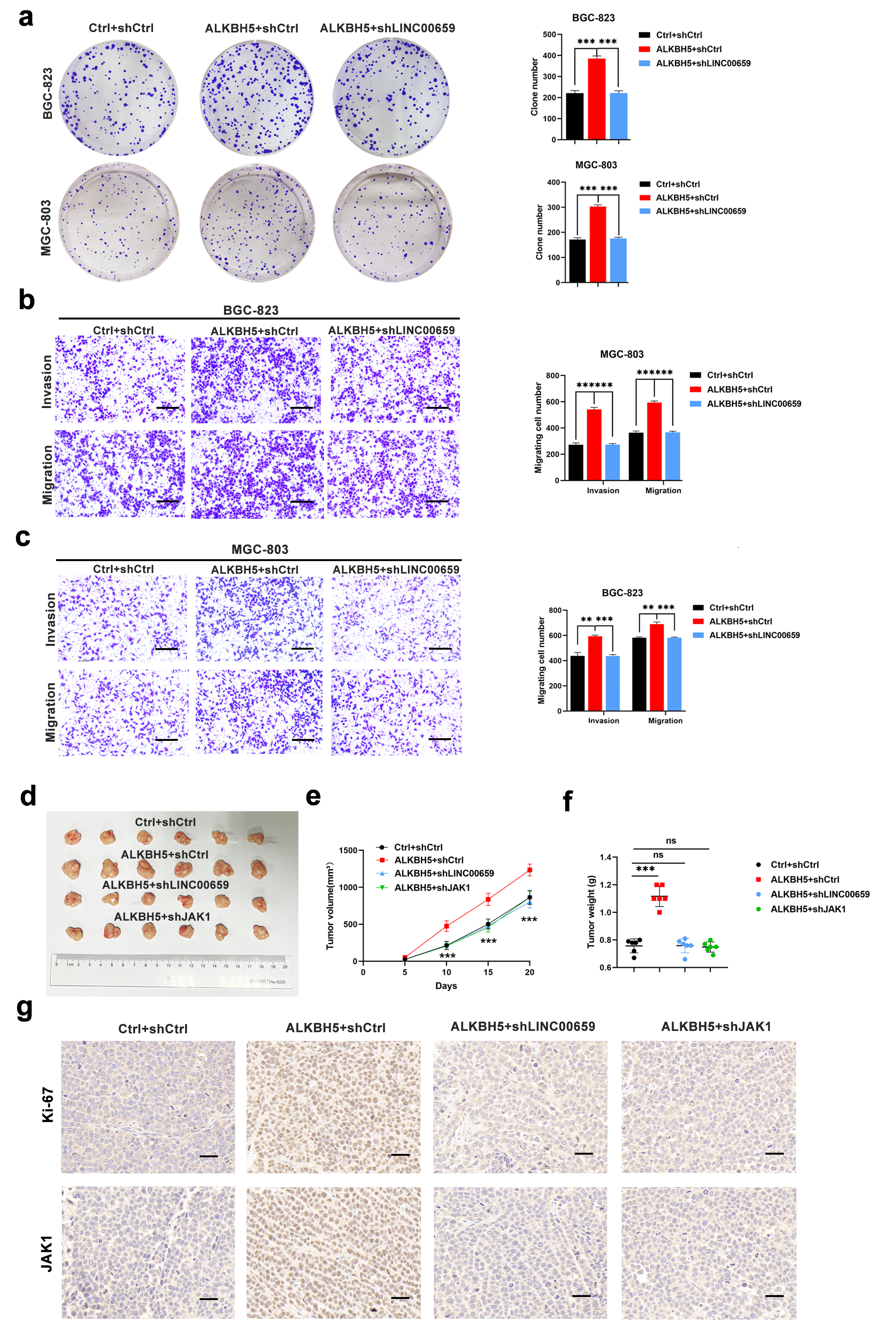

Supplement: Supplementary file 11 — Supporting Information [file CTM2-13-e1205-s014.tif]

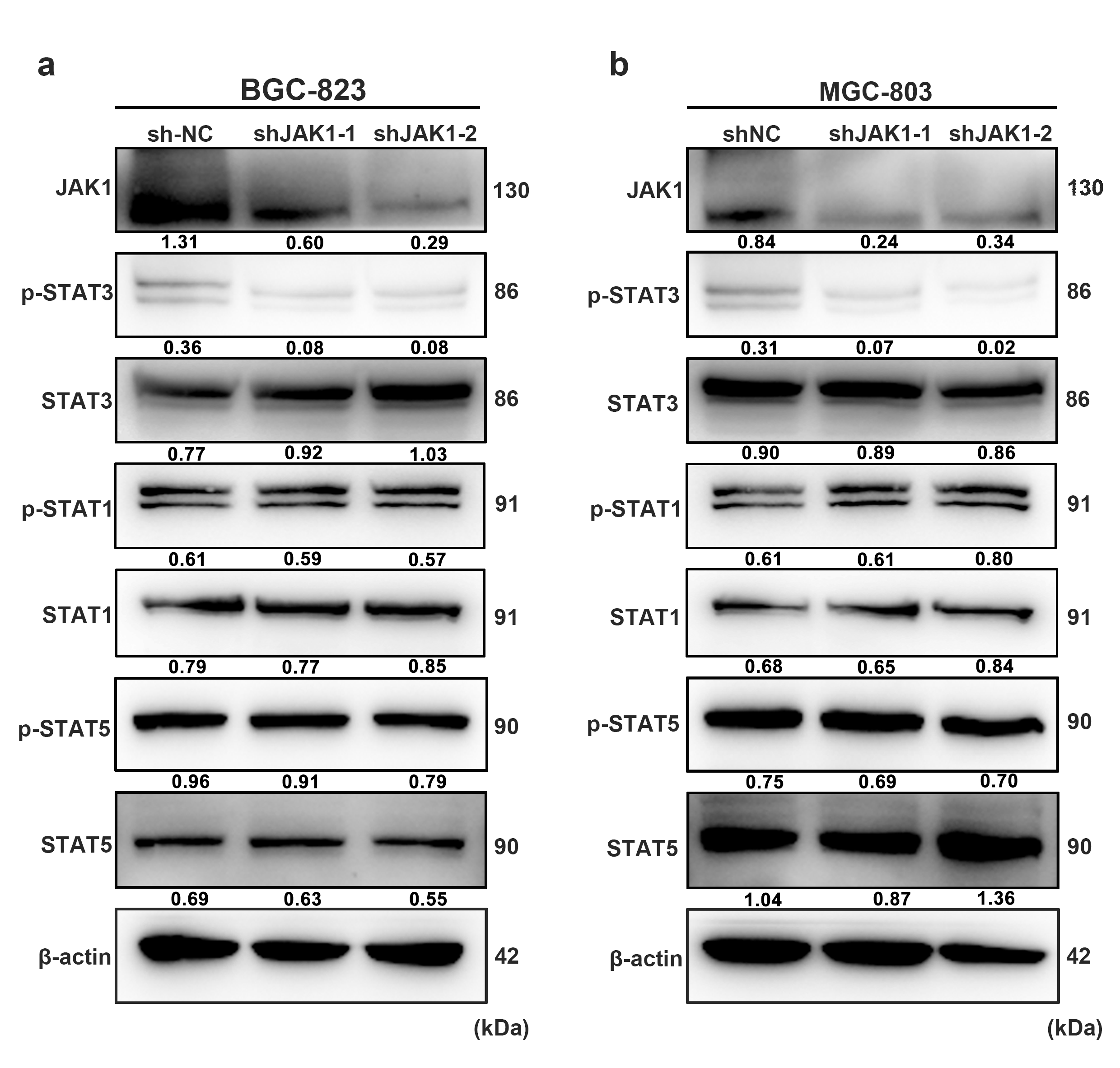

Supplement: Supplementary file 12 — Supporting Information [file CTM2-13-e1205-s004.tif]

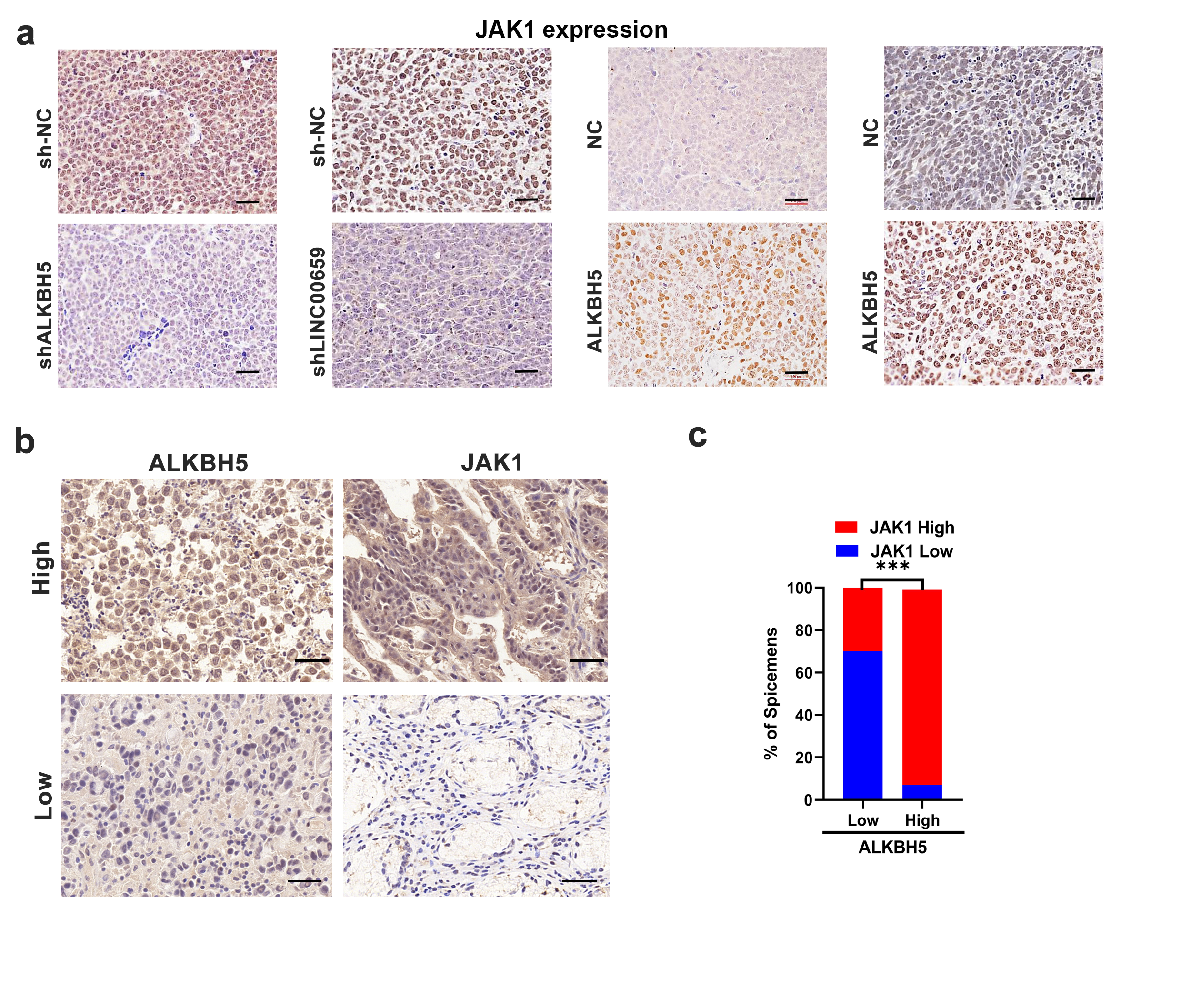

Supplement: Supplementary file 13 — Supporting Information [file CTM2-13-e1205-s002.tif]

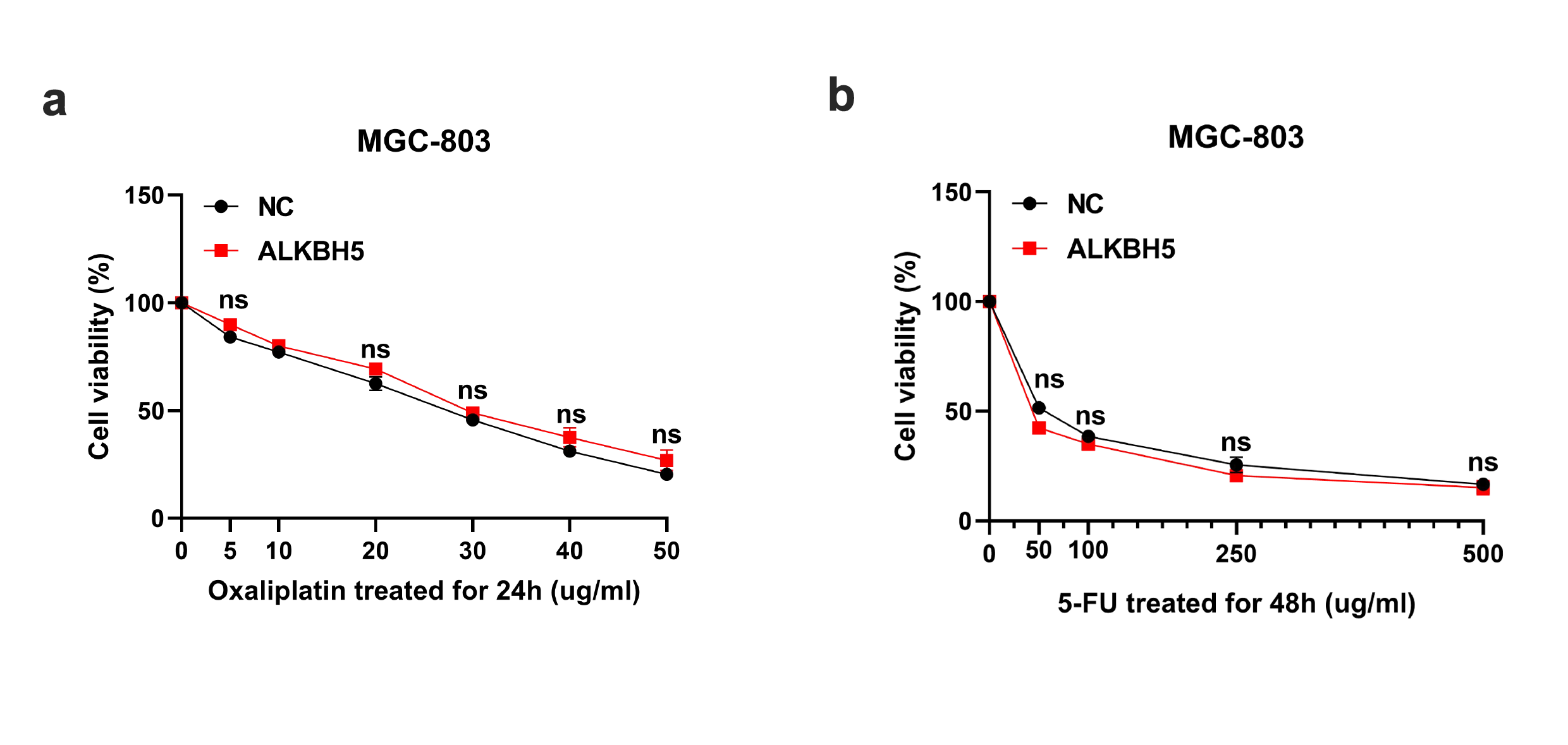

Supplement: Supplementary file 14 — Supporting Information [file CTM2-13-e1205-s007.tif]
