# Supplementary material for: LINC00659 cooperated with ALKBH5 to accelerate gastric cancer progression by stabilising JAK1 mRNA in an m6A‐YTHDF2‐dependent manner
Source: Clin Transl Med. 2023 Mar 2;13(3):e1205. doi: 10.1002/ctm2.1205 (PMC9982078; doi:10.1002/ctm2.1205)
Supplement: Supplementary file 15 — Supporting Information [file CTM2-13-e1205-s006.docx]

Supplementary Materials for

**ALKBH5/LINC00659/JAK1 axis promotes gastric cancer progression in a m6A-YTHDF2-dependent manner**

Yuan Fang^1, †^, Xi Wu^1, †^, Yunru Gu^1^, Run Shi^1^, Tao Yu^1^,Yutian Pan^1^, Jingxin Zhang^2^,Xinming Jing^1^, Pei Ma^1,*^ ,Yongqian Shu^1, 3,*^

Correspondence to: P Ma: mapei@njmu.edu.cn; Y Shu: yongqian_shu@163.com or shuyongqian@csco.org.cn

Materials and methods

**Cell transfection and lentiviral infection**

The siRNA targeting YTHDF2 was designed by RIBOBIO (Guangzhou, China). The lentivirus vector containing shRNAs targeting ALKBH5 or LINC00659 was generated by GenePharma (Shanghai, China). The lentivirus vector overexpressing LINC00659 was generated by HANBIO (Shanghai, China). The plasmid GV146, which overexpressed JAK1, and plasmid GV102, which knocked down JAK1, were purchased from GeneChem (Shanghai, China). We transfected plasmid vectors and siRNAs into cells with Lipofectamine 3000 (Invitrogen, USA) according to manufacturer’s introductions, and qRT-PCR and Western blotting assays were used to confirm the transfection efficiency. Lentivirus vectors were transfected into cells with polybrene, and stable cell lines were selected under 1 µg/ml puromycin condition. All the nucleotide sequences used to knockdown some gene expression are shown in **Table S1**.

**RNA extraction and quantitative real-time polymerase chain reaction (qRT-PCR)**

TRIzol reagents were purchased from Invitrogen(USA) and used to extracted the RNA from cells and tissues as directed by the manufacturer. A Reverse Transcription Kit (Invitrogen, USA) (Takara, Dalian, China) was used to reverse-transcribed RNA into cDNA for further analysis. Real-time PCR assays were performed using an ABI 7900HT real-time PCR system (Applied Biosystems, Canada). Table S1 lists the primers in order of their expression for all results.

**MeRIP assay and MeRIP-seq**

The commercial Magna MeRIP^TM^ m^6^A Kit (Millipore, USA) was used perform MeRIP assays according to the manufacturer’s protocol. Total RNA was fragmented in fragmentation buffer. Then, the m^6^A antibody and Magna ChIP Protein A/G Magnetic Beads were added to above buffer and rotated at room temperature for 30 minutes. Beads that captured the m^6^A antibody and MeRIP reaction mixture that contained fragmented RNA, RNase buffer and IP buffer Antibody were rotated for 2 hours at 4 ℃. The needed RNA was obtained from beads using elution buffer at 4 °C with continuous shaking for 1 hour. RNA was extracted and purified. Immunoprecipitated RNAs were analyzed using qRT-PCR or high-throughput sequencing. RNA library preparation and high-throughput sequencing were performed by RiboBio (Guangzhou, China). All of the primer sequences used in MeRIP are listed in Table S1.

**Luciferase Reporter Assays**

The luciferase reporter vector GV272 was inserted into mutant sequence or the wild-type sequence of m^6^A peak-enriched regions in JAK1 mRNA (GeneChem, China). We detected the luciferase activities by using the Dual-Luciferase® Reporter (DLR™) Assay System (Promega, USA, #E1910). HEK-293T cells (6×10^4^ cells/per well) were seeded in 24-well plates. Lipofectamine 3000 was used to co-transfect HEK-293T cells with plasmids with different sequences after 24 hours. After 24 h of incubation, a microplate reader (Synergy H1, USA) was used to detect luciferase activity. The plasmid sequences for luciferase reporter assays are listed in Table S1.

**Statistical Analysis**

GraphPad Prism 7 software (California, USA) was used for all statistical analyses. The in vitro and in vivo data were analyzed using Student’s t test (two-tailed), one-way analysis of variance, and the Spearman rank correlation. A 0.05 alpha level was used to determine the significance of each experiment, which was repeated at least three times. All data are presented as the means ± standard errors of the means (SEM).

| **Primers used for qRT-PCR** | **Sequence(5'-3')** |
| --- | --- |
| β-actin Forward | CATGTACGTTGCTATCCAGGC |
| β-actin Reverse | CTCCTTAATGTCACGCACGAT |
| ALKBH5 Forward | CGGCGAAGGCTACACTTACG |
| ALKBH5 Reverse | CCACCAGCTTTTGGATCACCA |
| LINC00659 Forward | ATGCTCGCCCATCTGTCG |
| LINC00659 Reverse | TCTCCGCTAATCCCACCC |
| LINC01123 Forward | ACAGTGGCCGCACGCATAGCTG |
| LINC01123 Reverse | CTGACGACCGAGGTGACAACGATGA |
| PVT1 Forward | CCTGGTGAAGCATCTGATGCACG |
| PVT1 Reverse | GCCAGGCTTTGTGGCACACGC |
| CLEC12A-AS1 Forward | TCAGATGAGATTGGGCATGTTCAG |
| CLEC12A-AS1Reverse | TTGGGTGTATTCTTCAGGGTTGG |
| JAK1 Forward | CACAGAAGACGGAGGAAA |
| JAK1 Reverse | CCGAGAACCCAAATAGTC |
| H19 Forward | TGCTGCACTTTACAACCACTG |
| H19 Reverse | ATGGTGTCTTTGATGTTGGGC |
| \| **siRNAs/shRNA** \| \| --- \| | **Targeting sequences** |
| shALKBH5-1 | 5‘-GGTTGGAAACAAAGTCCCTGA-3' |
| shALKBH5-2 | 5’-GCTTCAGCTCTGAGAACTACT-3' |
| shJAK1-1 | 5'-GACAGTCACAAGACTTGTGAA-3' |
| shJAK1-2 | 5'-CTTCGGTTTAACCAAAGCAAT-3' |
| shLINC00659-1 | 5'-GGCTTTGAGGTTGTGCACA-3' |
| shLINC00659-2 | 5'-GGATTAGCGGAGATCCACT-3' |
| siYTHDF2-1 | 5'-GCACAGAAGTTGCAAGCAA-3' |
| siYTHDF2-2 | 5'-GGTAGCGGGTCCATTACTA-3' |
| siH19 | 5-′CAGCCCAACATCAAAGACA-3′ |
| siPVT1 | 5-′GAGCUGCGAGCAAAGAUGUTT-3′ |
| siLINC01123 | 5-′CUGAACGUCUUGCAACAGUTT-3′ |
| siCLEC12A-AS1 | 5-′GCTGAACTTGCCAATGGAA-3′ |
| **Primers used for qRIP,qMeRIP** | **Sequence(5'-3')** |
| LINC00659 Forward | AACCCTGTGACCCAGAAACACCAAT |
| LINC00659 Reverse | GAGACACCCTGAATGACG |
| JAK1 Forward | CACAGAAGACGGAGGAAA |
| JAK1 Reverse | CCGAGAACCCAAATAGTC |
| LINC00659 Exon1-2 Forward | GGCACCCCTGAAGGACCA |
| LINC00659 Exon1-2 Reverse | CTCCCAAGAGAGGGGGAG |
| LINC00659 Exon3 Forward | GTAGAGAGCAGTGAGGAA |
| LINC00659 Exon3 Reverse | GACGGTCTTTTTCTTTTA |
| LINC00659 124-300bp Forward | GTAGAGAGCAGTGAGGAA |
| LINC00659 124-300bp Reverse | TCCAGACTTGGCGTGAAG |
| LINC00659 301-572bp Forward | AAAAATGGCTTTGAGGTT |
| LINC00659 301-572bp Reverse | GACGGTCTTTTTCTTTTA |
| **luciferase reporter assays** | **sequences** |
| JAK1-wt | 5'-TCACATTGTAATAAAGGAGTCTGTGGTCAGCATTAACAAGCAGGACAACA-3' |
| JAK1-mt | 5'-TCACATTGTAATAAAGGAGTCTGTGGTCAGCATTAACAAGCAGGCCAACA-3' |

**Table S1.Primers used for qRT-PCR, qRIP,qMeRIP and siRNAs/shRNA Targeting sequences**

| **Antibody** | **Host** | **Supplier** | **Catalog No.** | **Application** |
| --- | --- | --- | --- | --- |
| ALKBH5 | Rabbit | Abcam | ab195377 | WB,IHC,RIP |
| YTHDF2 | Rabbit | Abcam | ab220163 | WB,RIP |
| JAK1 | Mouse | Proteintech | 66466-1-lg | WB，IHC |
| STAT1 | Mouse | CST | 9H2 | WB |
| p-STAT1 | Rabbit | CST | D4A7 | WB |
| STAT3 | Mouse | CST | 124H6 | WB |
| p-STAT3 | Rabbit | CST | D3A7 | WB |
| STAT5 | Mouse | CST | ab230670 | WB |
| p-STAT5 | Rabbit | CST | D47E7 | WB |
| β-actin | Mouse | CST | 8H10D10 | WB |
| m^6^A | Mouse | Abcam | ab208577 | MeRIP |
| GAPDH | Mouse | CST | D4C6R | WB |

**Table S2. Antibodies**

| Term | Count | % | P Value | Fold Enrichment | FDR | Genes |
| --- | --- | --- | --- | --- | --- | --- |
| Signal Transduction | 12 | 30.76923077 | 0.020132626 | 1.947445343 | 1 | JAK1,AXL,FOS,  GPR39,RACGAP1  AMH,COL27A1,  LTBP1,NR4A1,  PDE7B,PCDH7,  RHOU |
| TGF-beta receptor signaling | 3 | 7.692307692 | 0.006650648 | 22.81034483 | 0.455744618 | JAK1,FOS,LTBP1 |
| TGF-beta receptor signaling in skeletal dysplasias | 3 | 7.692307692 | 0.007570082 | 21.33870968 | 0.455744618 | JAK1,FOS,LTBP1 |
| Th17 cell differentiation pathway | 3 | 7.692307692 | 0.009836215 | 18.63380282 | 0.455744618 | JAK1,FOS,LTBP1 |
| Interleukin-4 and Interleukin-13 signaling | 3 | 7.692307692 | 0.025235145 | 11.62606838 | 1 | JAK1,FOS,RHOU |

**Table S3. Enrichment analysis of 39 genes**
